# Safety and Antitumor Activity of the Usnic Acid Derivative OL9-116, a TDP1 Inhibitor, as an Adjunctive Therapy in Combination with Topotecan

**DOI:** 10.3390/ijms27209266

**Published:** 2026-10-19

**Authors:** Tatyana E. Kornienko, Alexandra L. Zakharenko, Irina A. Chernyshova, Arina A. Chepanova, Nadezhda S. Dyrkheeva, Alina A. Firsova, Artem D. Rogachev, Olga A. Luzina, Nelly A. Popova, Varvara P. Maksimova, Olga A. Vlasova, Valentina P. Deryagina, Alexander Ya. Rodnyy, Nariman F. Salakhutdinov, Olga I. Lavrik

**Affiliations:** 1 Knorre Institute of Chemical Biology and Fundamental Medicine, Siberian Branch of the Russian Academy of Sciences, 630090 Novosibirsk, Russia; t.kornienko1995@gmail.com (T.E.K.); arinachepanova@mail.ru (A.A.C.);; 2 Federal Research Center Institute of Cytology and Genetics, Siberian Branch of the Russian Academy of Sciences, 10, Akademika Lavrentieva Ave., 630090 Novosibirsk, Russia; 3 N. N. Vorozhtsov Novosibirsk Institute of Organic Chemistry, Siberian Branch of the Russian Academy of Sciences, 9, Akademika Lavrentieva Ave., 630090 Novosibirsk, Russiarogachev@nioch.nsc.ru (A.D.R.); luzina@nioch.nsc.ru (O.A.L.);; 4 Institute of Medicine and Medical Technologies, Novosibirsk State University, Pirogov St., 2, 630090 Novosibirsk, Russia; 5 Department of Natural Sciences, Novosibirsk State University, Pirogov St., 2, 630090 Novosibirsk, Russia; 6 N.N. Blokhin National Medical Research Center of Oncology, 24 Kashirskoe Shosse, 115478 Moscow, Russia; 7 The Institute of Biology and Biotechnology, Altai State University, Pr. Lenina 61, 656049 Barnaul, Russia

**Keywords:** TDP1 inhibitors, topotecan, combination chemotherapy, Ames assay, cytokinesis-block micronucleus (CBMN) assay, epigenetic effects, pharmacokinetics, Lewis lung carcinoma (LLC), cervical cancer (RShM-5)

## Abstract

Topotecan inhibits topoisomerase 1 (TOP1) by trapping the enzyme in a DNA-cleavage complex, which can be repaired by tyrosyl-DNA phosphodiesterase 1 (TDP1). The usnic acid derivative OL9-116 inhibits TDP1 and thereby enhances the antitumor activity of topotecan, making it a promising combination partner. However, the safety and optimal administration schedule of OL9-116 with topotecan have not been characterized. We demonstrated that OL9-116 has no genotoxic or epigenetic effects using Ames and CBMN assays, as well as HeLa trichostatin A-inducible cells with an epigenetically repressed GFP gene. Intraperitoneal administration was found to provide higher bioavailability than intragastric administration. We showed on the RShM-5 cervical cancer model that repeated topotecan administration in a clinically relevant low single-dose regimen was more effective than a single injection, and addition of topotecan at the T_max_ of OL9-116 enhanced tumor growth inhibition. In the Lewis lung carcinoma model, the OL9-116–topotecan combination reduced primary tumor mass and lung metastases, enhancing both antitumor and antimetastatic effects. Monitoring of hematological parameters, liver, and spleen indices revealed no clinically significant toxicity. Moreover, peripheral blood parameters improved with the OL9-116–topotecan combination compared with topotecan monotherapy. Collectively, these findings support OL9-116 as a promising safe TDP1 inhibitor for combination with topotecan.

## 1. Introduction

The development of combination therapy in oncology reflects the molecular complexity of tumor cells, the instability of their genomes, and the genetic and phenotypic heterogeneity of tumors. These factors underlie the rapid appearance of tumor resistance to drug treatment [1,2,3]. Treatment with a single agent rarely achieves durable and complete disease control, because a subset of tumor cells can survive due to pre-existing or therapy-induced resistance mechanisms. Therefore, combination chemotherapy is considered a more promising approach to enhancing the efficacy of anticancer treatment [4]. This therapy enables the tumor to be targeted simultaneously in several ways: suppressing proliferation, disrupting DNA repair, blocking growth-signaling pathways, inhibiting angiogenesis, and enhancing the immune response. This multi-targeted approach reduces the likelihood of resistant clones persisting and increases the probability of achieving a clinically meaningful response to treatment [5]. In clinical oncology, this is particularly important for metastatic tumors, for which monotherapy is often insufficient [6]. The key concept of combination therapy is the rational selection of drugs with synergistic or complementary effects that do not proportionally increase toxicity, rather than merely the concurrent administration of several agents [7,8,9,10]. This is particularly relevant for regimens in which the auxiliary agent is not the primary cytotoxic drug but instead facilitates its delivery to tumor cells, increases tumor cell sensitivity, or prevents the development of drug resistance. A classic example is the combination of chemotherapy with targeted agents that enhance the effect of the principal drug by inhibiting specific molecular targets [11]. Rationally combining a primary drug with an adjuvant that enhances its activity enables the realization of synergistic interactions, increases the efficacy and durability of the response, helps to overcome tumor resistance mechanisms, and can potentially reduce toxic burden through dose optimization. In this context, combination regimens represent one of the key strategies of personalized oncology.

In the development and preclinical evaluation of new compounds with potential antitumor activity, it is insufficient to focus solely on cytotoxic effects and the ability to suppress tumor cell proliferation, because the true therapeutic value of a drug is determined by both its efficacy and safety profile. At the preclinical stage, it is therefore essential to characterize the spectrum of potential adverse effects, including general toxicity, mutagenic and genotoxic activity, and possible epigenetic effects. A compound may fail to exhibit pronounced cytotoxicity in short-term assays yet still induce genomic instability, disrupt gene expression regulation, or alter DNA methylation patterns, histone modifications and non-coding RNA expressions. Therefore, comprehensive characterization of a promising compound in preclinical studies must encompass confirmation of its antitumor activity alongside an in-depth assessment of its toxicity, mutagenicity, clastogenicity, and epigenetic safety. Ultimately, it is the combination of high efficacy and an acceptable risk profile for diverse adverse effects that ultimately determines the feasibility of further development of a candidate medicinal product.

**Figure 1:**
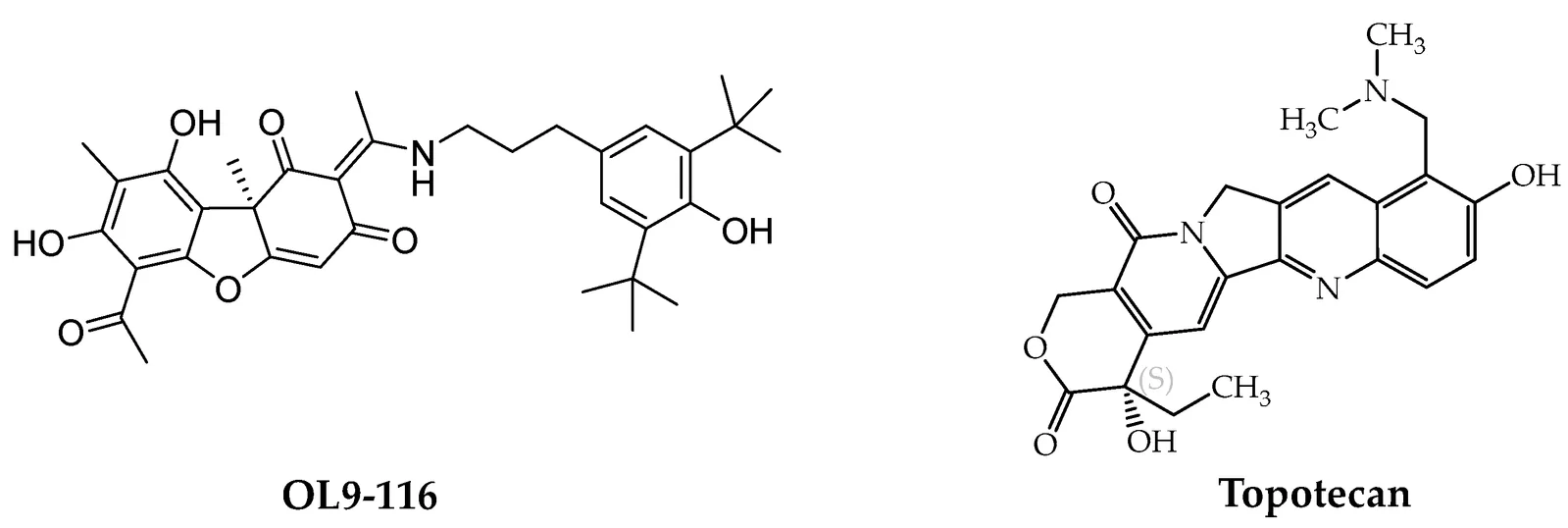
Chemical structures of OL9-116 (**left**) and topotecan (**right**).

We previously identified OL9-116, an enamine derivative of usnic acid, as a highly effective inhibitor of tyrosyl-DNA phosphodiesterase 1 (TDP1) [12,13] (Figure 1). TDP1 is considered a promising therapeutic target in oncology because it plays a key role in the removal of DNA lesions induced by antitumor agents that inhibit topoisomerase 1 (TOP1), such as topotecan and irinotecan [14,15]. Topotecan is approved by the Ministry of Health of the Russian Federation for the treatment of several malignancies, but it is associated with serious hematological and gastrointestinal toxicity. Its cytotoxic action is mediated by the inhibition of TOP1, which leads to the formation of covalent TOP1–DNA adducts and subsequent arrest of DNA replication, and, consequently, cell proliferation is inhibited. Using TDP1 inhibitors to enhance the efficacy of topotecan could reduce the therapeutic dose and attenuate treatment-related toxicity. Topotecan (Hycamtin) is FDA-approved in two dosage forms: an intravenous formulation (since 1996, NDA 020671) for the treatment of metastatic ovarian cancer, small-cell lung cancer, and cervical cancer, and oral capsules (since 2007, NDA 020981) for the treatment of relapsed small-cell lung cancer.

It has been shown that OL9-116 exhibits no significant toxicity in cell cultures or laboratory animals, while demonstrating a notable ability to enhance the antitumor activity of topotecan both in vitro and in vivo [12,16]. Subsequent studies demonstrated that using OL9-116 in combination with topotecan in a mouse model of Lewis lung carcinoma (LLC) significantly reduced tumor volume and decreased metastatic activity. A similar sensitizing effect was confirmed in the Krebs-2 ascitic carcinoma model: treatment with topotecan combined with OL9-116 resulted in a significant reduction in ascitic fluid volume and a decrease in the total number of viable tumor cells in the exudate [13]. The compound OL9-116 and its combination with topotecan are well tolerated by laboratory animals and do not lead to a significant decrease in body weight, behavioral changes, or alterations in organ indices [12,13]. A pharmacokinetic analysis of OL9-116 administered intragastrically at a dose of 150 mg/kg to mice was performed using whole blood, organ tissue, and tumor tissue samples [17,18]. The obtained data made it possible to determine whether the T_max_ of the compound in the blood is reached and to increase the efficacy of the combination therapy of topotecan and OL9-116 in an experimental in vivo Lewis lung carcinoma model by temporal alignment of the maximum concentrations (C_max_) of OL9-116 and topotecan administration [17].

The present study aims to evaluate the safety and therapeutic efficacy of the compound OL9-116. The objectives of the work include characterization of its biological safety profile, encompassing assessment of genotoxic and mutagenic potential, as well as identification of possible epigenetic effects of this compound. Another key goal is to develop and extrapolate a clinically relevant course-dosing regimen of topotecan for use in experimental mouse models in vivo, while also investigating the pharmacokinetics of OL9-116 following intraperitoneal administration. Based on the pharmacokinetic data obtained, the study aims to substantiate a combination therapy regimen that couples topotecan with OL9-116 in order to reduce drug dose while maintaining antitumor and antimetastatic efficacy.

## 2. Results and Discussion

### 2.1. Assessment of the Carcinogenic Safety of OL9-116 by Analyzing Its Mutagenic Properties Using a Modified Ames Assay In Vitro

We demonstrated that OL9-116 lacked mutagenic activity in the micro- and submicromolar concentration range in the experiments on the induction of reversions in *Salmonella typhimurium* in a modified Ames assay using strains TA98 and TA100. Exposure of the bacteria to the compound did not induce base pair substitutions or frameshift mutations in either the presence or absence of metabolic activation by the liver S9 fraction (Table 1).

The threshold criterion was a ≥2-fold increase in the number of colonies on test-substance plates versus the negative control.

### 2.2. Cytokinesis-Block Micronucleus Analysis of Human Lymphocytes for the Assessment of the Cytotoxicity and Genotoxicity of OL9-116

The cytokinesis-block micronucleus (CBMN) assay in human lymphocytes is used as a highly sensitive and comprehensive in vitro method that allows the simultaneous quantification of DNA damage (micronuclei, nucleoplasmic bridges and nuclear buds), cytostasis and cytotoxicity of the tested drug substances within a single system. Genotoxicity screening is usually aimed at identifying the clastogenic and aneugenic potential of chemical and pharmaceutical compounds. Using primary human lymphocytes with a normal mitotic apparatus increases the predictive value of the results for humans, and complies with current international requirements for genotoxicity screening [19,20].

To detect micronuclei or chromosomal aberrations, cell division is a prerequisite, and when performing the in vitro micronucleus assay with the addition of cytochalasin B, binucleated cells are observed. Therefore, the detection and testing of chromosomal aberrations require cells to be actively dividing and for the CBPI to exhibit high values. CBPI depends on the duration of cytochalasin B treatment, so there is no single, generally accepted standard value to ensure a good state of division. Generally, a value of at least 1.5 indicates that cells are sufficiently suitable for practical use as a detector and test system [21].

Table 2 presents the distribution of the lymphocyte population by number of nuclei (N1–N4), the cytokinesis-block proliferation index (CBPI), the frequency of micronucleated cells (MNed cells), and the numbers of nucleoplasmic bridges (NPBs) and nuclear buds (NBUDs) after exposure to the solvent (DMSO), two positive controls (IFO and Dac), and OL9-116 at four concentrations.

The total number of scored cells is comparable in all experimental groups, ensuring the validity of comparisons of damage frequencies across the different treatment conditions. IFO and Dac significantly increased the frequencies of MNed cells, NPBs and NBUDs compared with DMSO and all concentrations of OL9-116 in both donors (** *p* < 0.01, Pearson’s χ^2^ test).

The CBPI values for the DMSO control and all concentrations of OL9-116 were virtually identical (donor 1: 1.523 in the DMSO control versus 1.516–1.531 at OL9-116 concentrations of 0.1–100 µM; donor 2: 1.51 in the DMSO control versus 1.508–1.53 at OL9-116 concentrations of 10^−7^–10^−4^ M). CBPI values ranging from 1.5 to 1.6 are characteristic of actively proliferating lymphocyte cultures after stimulation with phytohemagglutinin [19]. These fluctuations can be regarded as statistically insignificant and within the range of natural variability of CBPI between independent cultures. The absence of a systematic decrease in CBPI with increasing OL9-116 concentration indicates a lack of pronounced cytostatic or cytotoxic activity of this compound within the tested concentration range. The positive controls demonstrated a moderate increase in CBPI relative to the vehicle control (donor 1: up to 1.604 for IFO and 1.669 for Dac; donor 2: up to 1.609 for IFO and 1.649 for Dac), accompanied by a reduction in the proportion of mononucleated lymphocytes (N1) and an increase in the proportion of multinucleated cells (N ≥ 3). For CBPI and the distribution of nuclei per cell (N1/N2/N ≥ 3), a significant difference was observed only for Dac in both donors (** *p* < 0.01); IFO reached *p* < 0.05 in donor 2 only and was not significant in donor 1 (compared with DMSO and all concentrations of OL9-116). This reflects enhanced proliferative activity under genotoxic stress, rather than toxic inhibition of cell division.

#### 2.2.1. Micronucleus Induction

In the control cultures of donors 1 and 2 that were incubated with DMSO, the frequency of cells with micronuclei per 1000 binucleated cells was found to be 11.5 and 10.5, respectively (Table 2), which corresponds to the spontaneous background level of chromosomal damage. Exposure to IFO increased the micronucleus frequency to 40.5 per 1000 cells in the lymphocyte culture of donor 1 (81 cells with ≥1 micronucleus per 2000 cells) and to 46 per 1000 cells in the lymphocyte culture of donor 2 (92 cells with ≥1 micronucleus per 2000 cells). Treatment with Dac resulted in values of 37 per 1000 cells in the lymphocyte culture of donor 1 (74 cells with ≥1 micronucleus per 2000 cells) and 41.5 per 1000 cells in the lymphocyte culture of donor 2 (83 cells with ≥1 micronucleus per 2000 cells). This result corresponds to a 3–4-fold increase in micronucleus frequency relative to the solvent control and represents a statistically significant elevation (*p* = 0.01). Thus, it confirms the adequacy and sensitivity of the test system.

Micronucleus frequencies remained comparable to the DMSO-treated control across all OL9-116-treated groups, with no discernible concentration-dependent trend over the 0.1–100 µM range. The similarity of micronucleus frequencies in treated and control cultures, together with the lack of a dose-related response, indicates that OL9-116 does not cause a statistically significant increase in chromosomal damage in human lymphocytes from either male or female donors at the tested concentrations.

#### 2.2.2. Nucleoplasmic Bridges (NPBs) and Nuclear Buds (NBUDs)

In the DMSO-treated control groups of both donors, the frequencies of NPB and NBUD were minimal, which is consistent with a state of genomic stability in the absence of genotoxic exposure (Table 2). Upon exposure to IFO and Dac, a significant increase in the number of NPBs and NBUDs was observed, with the effect being particularly pronounced under Dac treatment. Donor 1 exhibited 25 NPBs and 45 NBUDs following IFO treatment, whereas 45 NPBs and 48 NBUDs were detected following Dac. Donor 2 showed a similar pattern: 32 NPBs and 51 NBUDs with IFO, increasing to 49 NPBs and 67 NBUDs with Dac. These values exceed those of the negative control (DMSO), where only 1–2 NPBs and 2 NBUDs were observed, by more than an order of magnitude, consistent with enhanced chromosomal instability and increased DNA breakage and error-prone replication processes.

Across all OL9-116 treatment conditions, the frequency of nucleoplasmic bridges ranged from zero to two events per sample, and the frequency of nuclear buds ranged from zero to three, remaining at or within the range of fluctuations of the control values, with no systematic increase as the compound concentration rose. The lack of an increase in nucleoplasmic bridges and nuclear buds further indicates that OL9-116 does not appreciably induce chromosomal instability or lead to the formation of large DNA extrusions or incomplete replication events.

Overall, the stable CBPI values, the absence of any dose-dependent increase in micronucleus frequency, and the extremely low levels of nucleoplasmic bridges and nuclear buds at all OL9-116 concentrations examined indicate that this compound does not have cytotoxic or genotoxic effects in vitro on lymphocytes from either donor. In parallel, the significant induction of micronuclei and chromosomal instability structures by IFO and Dac compared to DMSO validates the experimental design and demonstrates the adequate sensitivity of the assay in detecting genotoxic DNA damage.

Thus, in peripheral blood lymphocyte cultures from both male and female donors, OL9-116, over the concentration range of 0.1–100 µM, showed no evidence of clastogenic or aneugenic activity. Although 2000 diploid cells were analyzed in each case, the biological representativeness of the study is limited to two donors. Therefore, we do not draw conclusions regarding the complete safety of OL9-116 for the human genome, but we consider the obtained data encouraging.

### 2.3. OL9-116 Does Not Reactivate the Epigenetically Silenced GFP Reporter in HeLa TI Cells

HeLa TI (HeLa trichostatin A-inducible) cells represent a cellular reporter test system for assessing the epigenetic activity of xenobiotics and identifying agents that induce stable transcriptional shifts [22,23]. A distinctive feature of this test system is its sensitivity to a broad spectrum of epigenetic modulators, including HDAC (histone deacetylase), DNMT (DNA methyltransferase), HMT (histone methyltransferase) and BET (bromodomain and extra-terminal) inhibitors, as well as chromatin remodelers [22]. This sensitivity is driven by the involvement of more than 15 epigenetic silencing factors in maintaining the repressed state of the GFP reporter gene [24].

**Figure 2:**
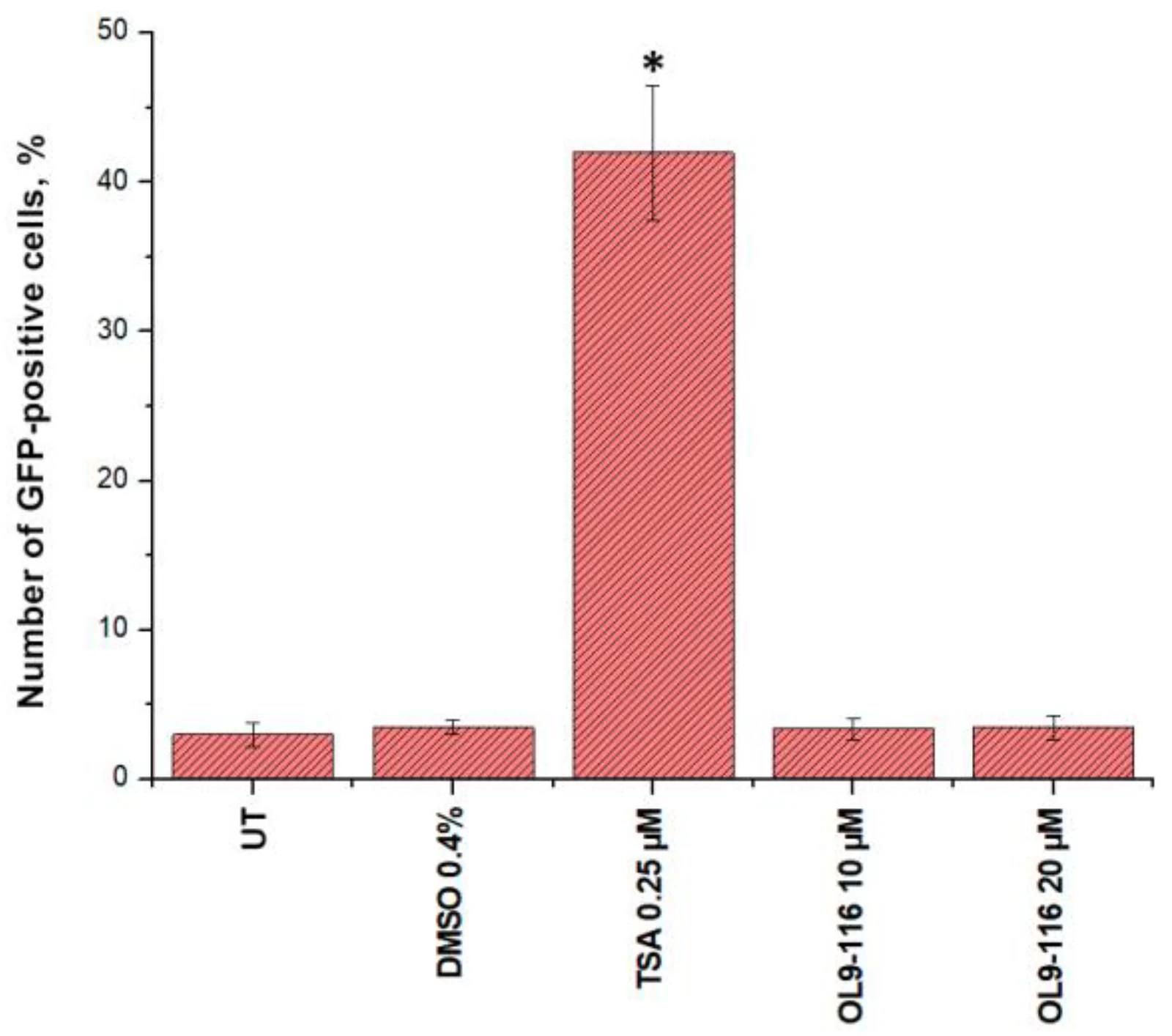
Reactivation of the epigenetically repressed GFP gene in HeLa TI cells following treatment with OL9-116. The graph shows the percentage of GFP-positive cells in the analyzed cell population. Trichostatin A (TSA) was used as a positive control. Data are presented as mean ± SD (n ≥ 3). * *p* < 0.05 compared to vehicle control (DMSO). UT—untreated cells.

It has been shown that exposure to OL9-116 did not result in a statistically significant increase in the number of cells with a GFP-positive phenotype, indicating preservation of epigenetic silencing of the reporter gene (Figure 2). Thus, OL9-116 does not exhibit epigenetic activity.

### 2.4. Extrapolation of the Clinically Used Course Regimen of Topotecan Administration to In Vivo Therapy of the RShM-5 Tumor Model in Mice Using an Interspecies Dose Conversion Factor

Extrapolation of clinical course-based dosing regimens of anticancer drugs to murine tumor models using interspecies dose-conversion coefficients is necessary to approximate drug exposure in animals as closely as possible to real-world therapeutic conditions in patients, thereby enhancing the translational value of the resulting efficacy and toxicity data. This approach enables a scientifically sound justification for dose selection and administration schedules in in vivo experiments and facilitates interpretation of the results in the context of clinical application. The concept of interspecies dose extrapolation is based on the premise that the effects of many agents are largely determined by body mass, body surface area, and basal metabolic rate, which differ systematically between species. To account for these differences, allometric approaches are widely used, including body surface area-based dose conversion (mg/m^2^) using species-specific coefficients and/or power functions of body mass. Such methods are recommended by regulatory authorities and toxicology guidelines for translating data between laboratory animals and humans when evaluating safe and efficacious exposure levels [25,26,27,28].

In our previous studies using murine models of Krebs-2 ascites carcinoma and Lewis lung carcinoma (LLC) [13], we demonstrated that a single intraperitoneal administration of topotecan (TPC) at a dose of 1 mg/kg resulted in a statistically significant reduction in tumor mass compared to control animals that did not receive therapy. In the present study, we extrapolated the clinical course-based dosing regimen of TPC to the RShM-5 mouse model in vivo using an interspecies dose-conversion coefficient. This was done to align the experimental conditions as closely as possible with clinical practice, and to evaluate the antitumor activity of topotecan under a more clinically relevant dosing schedule.

**Figure 3:**
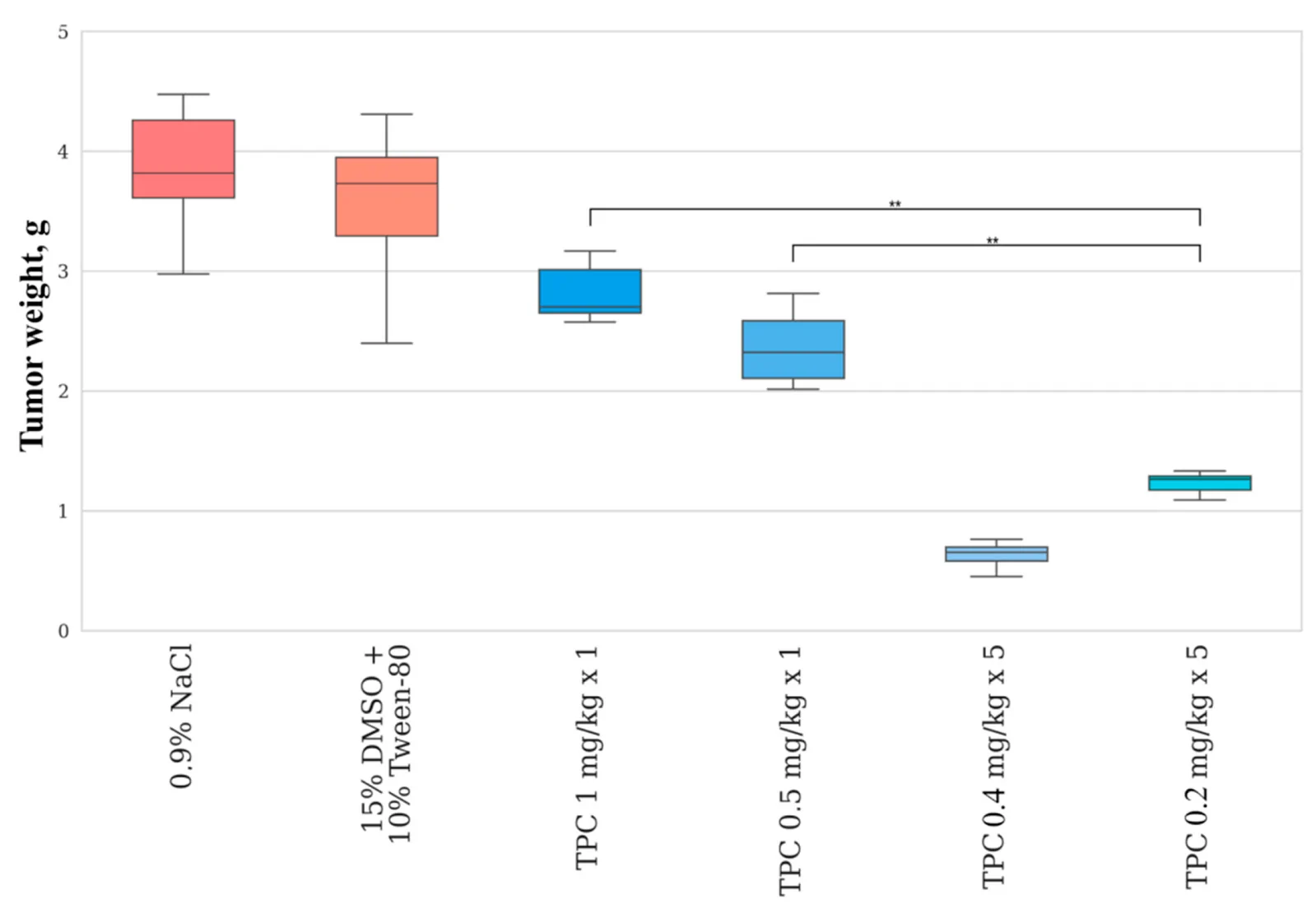
Effect of different doses and administration schedules of TPC on the weight of the primary RShM-5 tumor node. ** *p*-value < 0.001.

Figure 3 shows the mean weight of the primary tumor nodule in mice that received TPC (against a background of 15% DMSO and 10% Tween-80, the vehicle used for the Tdp1 inhibitors) at doses of 1 mg/kg as a single administration, 0.5 mg/kg administered twice, or 0.4 mg/kg or 0.2 mg/kg administered five times. As can be seen in Figure 3, dose fractionation led to an increase in therapeutic efficacy. Treatment with TPC at a dose of 0.4 mg/kg for five consecutive days, analogous to the clinical regimen in humans, resulted in a sixfold reduction in tumor nodule weight, from 3.5 g to 0.6 g. Tumor nodules weighing less than 1 g are extremely difficult to handle, and further improvement in the tumor growth inhibition (TGI) parameter is difficult to achieve under these conditions. Therefore, for subsequent experiments we selected a TPC dose of 0.2 mg/kg administered five times, yielding a mean nodule weight of 1.2 g.

It should be noted that a dose of 0.2 mg/kg administered five times (total cumulative dose 1 mg/kg) was substantially more effective than the same dose administered as a single injection or split into two administrations (*p*-value < 0.001, Figure 3): the mean tumor nodule weight (TGI) was 1.2 g (65%), 2.8 g (21%), and 2.3 g (34%), respectively. Such dose fractionation leads to reduced toxicity and increased efficacy [29]. In our study, no body-weight loss or animal mortality was observed, whereas the improvement in therapeutic efficacy was pronounced.

### 2.5. Pharmacokinetic Study of OL9-116 in the Blood of Healthy Mice Following Intraperitoneal Administration

In a previous study [17], we evaluated the pharmacokinetics of OL9-116 after an intragastric administration to mice at a dose of 150 mg/kg using a validated LC-MS/MS method for quantification of the compound. The maximum concentration (C_max_) of OL9-116 in mouse blood was observed 3–4 h post-dose and was approximately 5000 ng/mL. Following reaching C_max_, OL9-116 blood levels declined monotonically; however, 24 h after dosing, the blood concentration of OL9-116 remained relatively high, at around 500 ng/mL.

**Figure 4:**
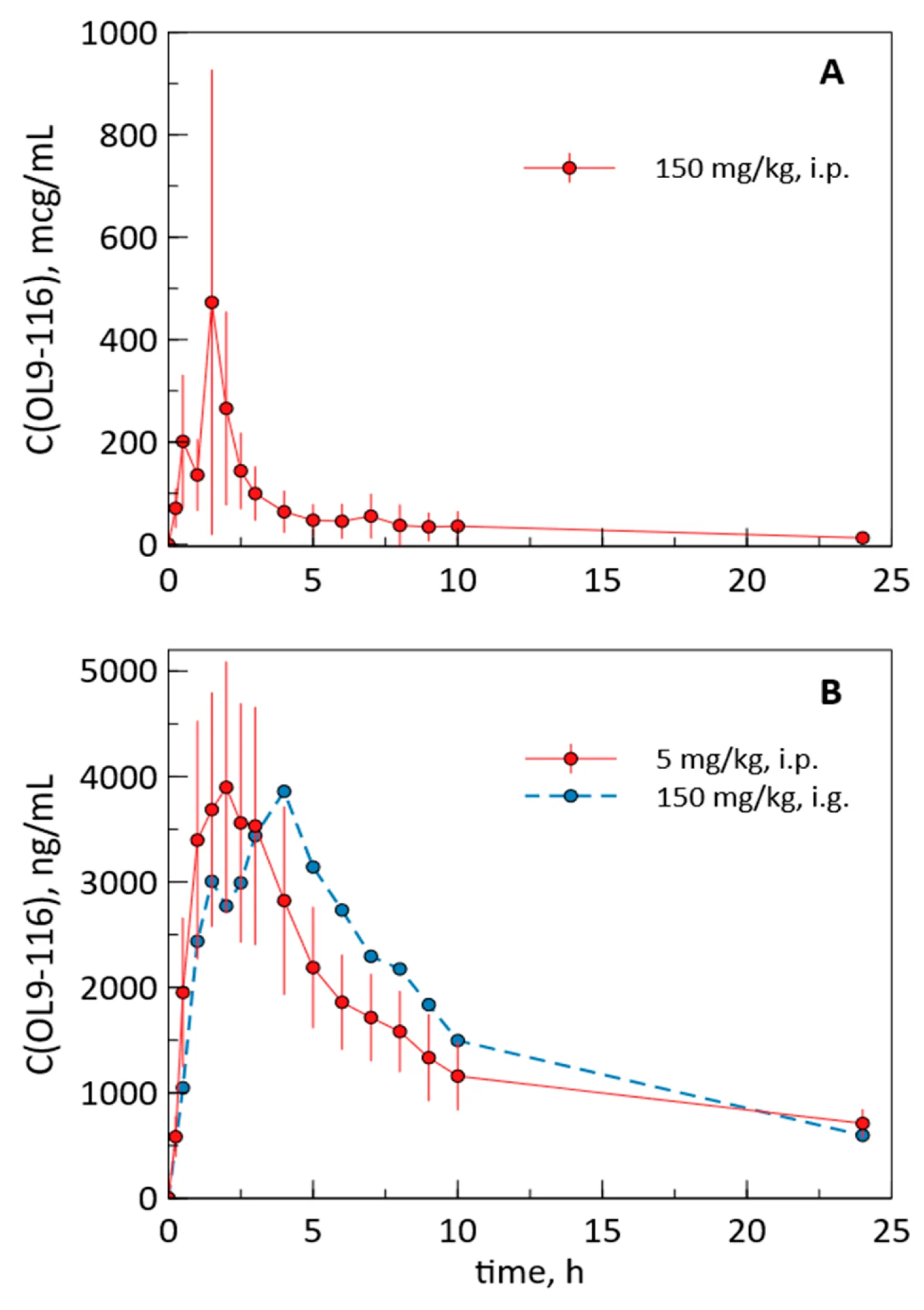
Concentration–time profile of OL9-116 in mouse blood following intraperitoneal administration: (**A**) at a dose of 150 mg/kg; (**B**) at a dose of 5 mg/kg. Data are presented as mean ± standard deviation (SD). For reference, panel (**B**) also shows the concentration–time profile after intragastric administration at a dose of 150 mg/kg (adapted from [17]).

In the present study, we investigated the pharmacokinetics of OL9-116 following intraperitoneal administration to mice at a dose of 150 mg/kg. Following intraperitoneal injection at a dose of 150 mg/kg, the maximum concentration of OL9-116 in blood was reached 2 h post-injection and was estimated to be at least 0.5 mg/mL (500 µg/mL, Figure 4A). These findings suggest that OL9-116 has substantially higher bioavailability after intraperitoneal administration, allowing for dose reduction.

Based on the above data, we conducted a pharmacokinetic study of OL9-116 in mouse blood following the agent’s intraperitoneal administration at a reduced dose of 5 mg/kg. Analysis of blood samples showed that under these conditions the T_max_ of OL9-116 was also 2 h; however, it was 1–2 h lower than the value observed after intragastric administration at a dose of 150 mg/kg (Figure 4B).

Comparing the two pharmacokinetic profiles and calculated parameters for intragastric administration at a dose of 150 mg/kg and intraperitoneal administration at a dose of 5 mg/kg, it should be noted that the maximum concentration reached 2 and 3 h after administration of the suspensions in the intragastric and intraperitoneal groups, respectively. Under these conditions, the maximum blood concentration of OL9-116 achieved after intraperitoneal administration of the reduced dose was virtually identical to that observed following intragastric administration at 150 mg/kg. The areas under the curve (AUC) for the two mean pharmacokinetic profiles were similar in order of magnitude, but higher for intraperitoneal administration: (6.4 ± 2.3) × 10^4^ ng/mL × h for the latter (Table S1 in Supplementary) and (5.0 ± 0.7) × 10^4^ ng/mL × h for intragastric administration at a dose of 150 mg/kg [17]. Based on the obtained AUC values and doses, it can be determined that intraperitoneal administration of the substance results in a more than 20-fold increase in its bioavailability. Thus, intraperitoneal administration of OL9-116 was found to markedly increase its bioavailability and to enable a 30-fold dose reduction.

### 2.6. Investigation of the Antitumor Activity of OL9-116 in Combination with Topotecan in the Cervical Carcinoma Model (RShM-5) Under a Course-Based Combined Administration Regimen with Synchronization of the Time of the Compound’s Maximum Blood Concentrations

The aim of the experiment was to investigate the effect of OL9-116 on the antitumor activity of TPC during course-based combined administration in the cervical cancer model (RShM-5) under conditions where the times to reach the maximum blood concentrations of both agents coincided, according to our calculations. According to published data, following the intraperitoneal administration of TPC at a dose of 10 mg/kg, the maximal blood concentration was achieved within the first few minutes (less than 5 min), followed by a rapid decline in drug levels, as confirmed by mathematical modeling of TPC pharmacokinetics and toxicodynamics in mice [30]. Although we did not measure the exact T_max_ of topotecan at a dose of 1 mg/kg, data from [17] indicate that sufficiently high concentrations of OL9-116 are maintained in the blood for a considerable time (about one hour); therefore, we believe the T_max_ of topotecan falls within this interval. Consequently, it can be expected that the peak blood concentrations of both substances are at least close to being synchronized when topotecan is administered two hours after OL9-116.

Considering our previous data on extrapolating the clinical TPC dosing regimen to the RShM-5 cervical cancer model (Figure 3) and the pharmacokinetic characteristics of OL9-116 following intraperitoneal administration (Figure 4), the present study evaluated the possibility of reducing the dose of the principal drug, TPC, upon incorporation of OL9-116 into the treatment regimen. In addition, we compared the antitumor efficacy and toxicity profile of the proposed combined regimen with previously employed approaches and dosing schedules of TPC and OL9-116.

#### 2.6.1. Effect of OL9-116 on RShM-5 Tumor Growth

**Figure 5:**
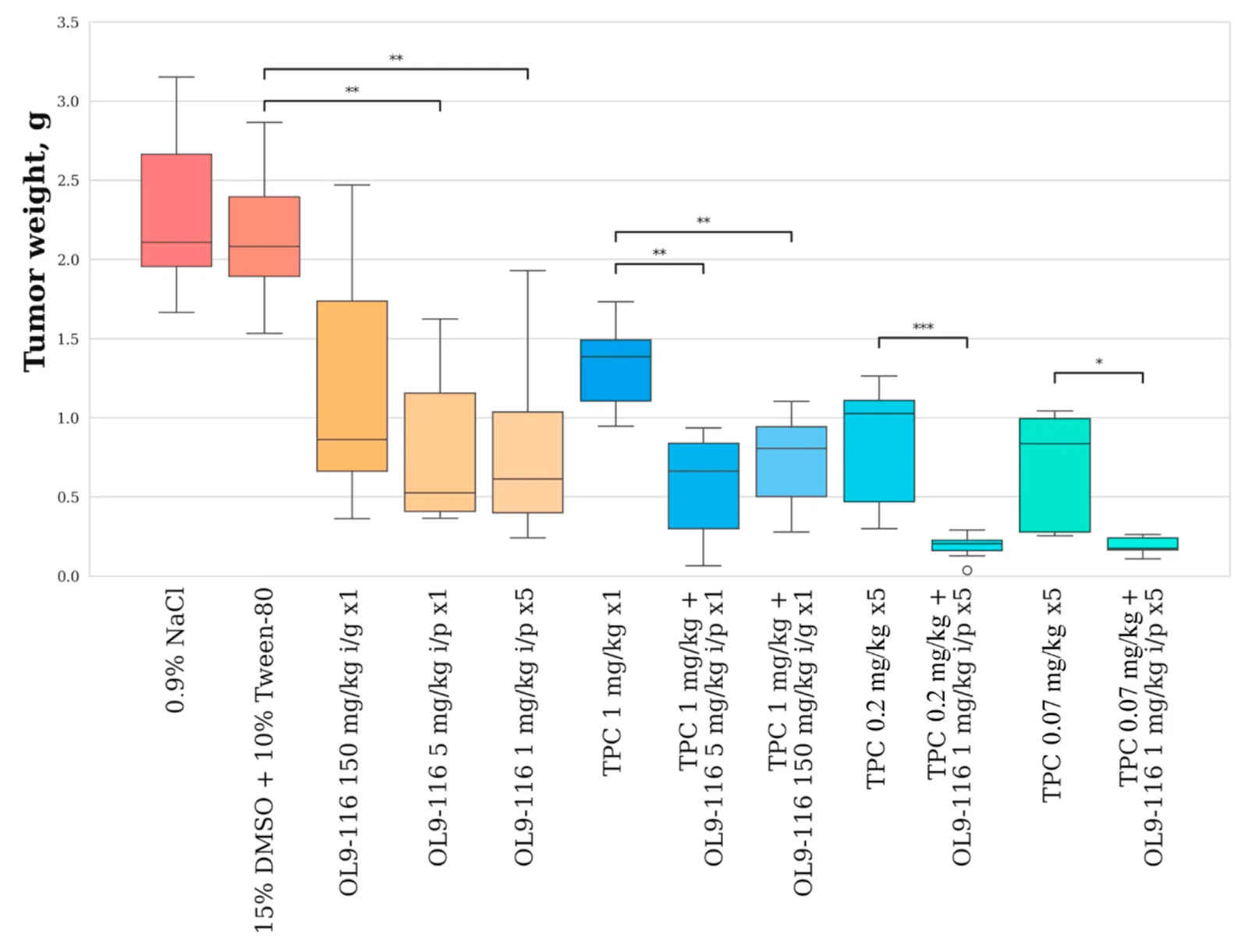
Effects of topotecan (TPC), OL9-116, and their combination, administered using different routes and dosing regimens, on primary tumor weight in RShM-5 cervical cancer-bearing mice. * *p* < 0.05, ** *p* < 0.01 and *** *p* < 0.001, Mann–Whitney U test. The number of animals was n = 7 per group.

In the control animal groups that received either 0.9% NaCl or the vehicle (15% DMSO + 10% Tween-80), rapid progression of the RShM-5 tumor growth was observed. The mean tumor nodule mass at the end of the experiment was 2.3 g in the group treated with normal saline and 2.1 g in the group treated with the vehicle; the TGI value for the vehicle group was only 7.4%, and the differences between the control groups were not statistically significant (*p* > 0.05, Figure 5). The low TGI value, combined with the absence of significant differences in tumor nodule mass, indicates a lack of any intrinsic clinically meaningful antitumor effect of the vehicle used, supporting its suitability as an inert carrier in this experimental model.

Administering OL9-116 as monotherapy intraperitoneally at a dose of 1 mg/kg once daily for five consecutive days or as a single 5 mg/kg dose resulted in only moderate inhibition of tumor growth compared to the control groups, with identical TGI values of 62%. A single intragastric (oral gavage) administration of OL9-116 at a dose of 150 mg/kg was less effective in reducing the primary tumor mass (TGI 44%), likely due to variability in compound absorption associated with this route of administration.

Single intraperitoneal administration of TPC at a dose of 1 mg/kg, which was previously used in our experiments involving models of ascitic Krebs-2 carcinoma and Lewis lung carcinoma (LLC) [13], resulted in a statistically significant reduction in tumor mass compared to the control group (TGI 43%). In contrast, a course of TPC administration at single doses of 0.2 and 0.07 mg/kg for five consecutive days was associated with a more pronounced tumor growth inhibition: the mean tumor nodule masses were comparable in both groups, while the TGI values were 64% and 70%, respectively. Thus, a course regimen of TPC given in a low single dose produced a more pronounced antitumor effect than a single injection at 1 mg/kg, which is consistent with published data demonstrating the strong dependence of TPC efficacy on the dosing schedule and the duration of drug exposure [29].

The combined administration of OL9-116 with a single 1 mg/kg dose of TPC resulted in a further statistically significant reduction in tumor mass compared to TPC monotherapy. The most effective suppression of tumor growth was observed following the intraperitoneal administration of OL9-116 at 5 mg/kg in combination with TPC at 1 mg/kg one time (TGI 73%), whereas a higher intragastric dose of 150 mg/kg in combination proved to be less effective (TGI 66%), despite the greater amount of compound administered. These findings suggest that intraperitoneal administration of OL9-116 results in more predictable systemic exposure and higher bioavailability than intragastric administration. This creates optimal conditions for OL9-116 to realize its sensitizing effect on the cytotoxic activity of TPC. Therefore, using OL9-116 in a parenteral dosing regimen appears to be preferable for enhancing the antitumor efficacy of TPC.

The most pronounced inhibition of tumor growth was observed in groups that received a combined course therapy, when TPC was administered at doses of 0.2 or 0.07 mg/kg in combination with OL9-116 at 1 mg/kg intraperitoneally for five consecutive days. The TGI value was 91% in both groups, with a median tumor mass of 0.1–0.3 g. These values were significantly lower than those observed in the corresponding TPC monotherapy groups (*** *p*-value < 0.001 and * *p*-value < 0.05, respectively).

#### 2.6.2. Effect of OL9-116 on the State of Peripheral Blood in Mice with RShM-5

**Figure 6:**
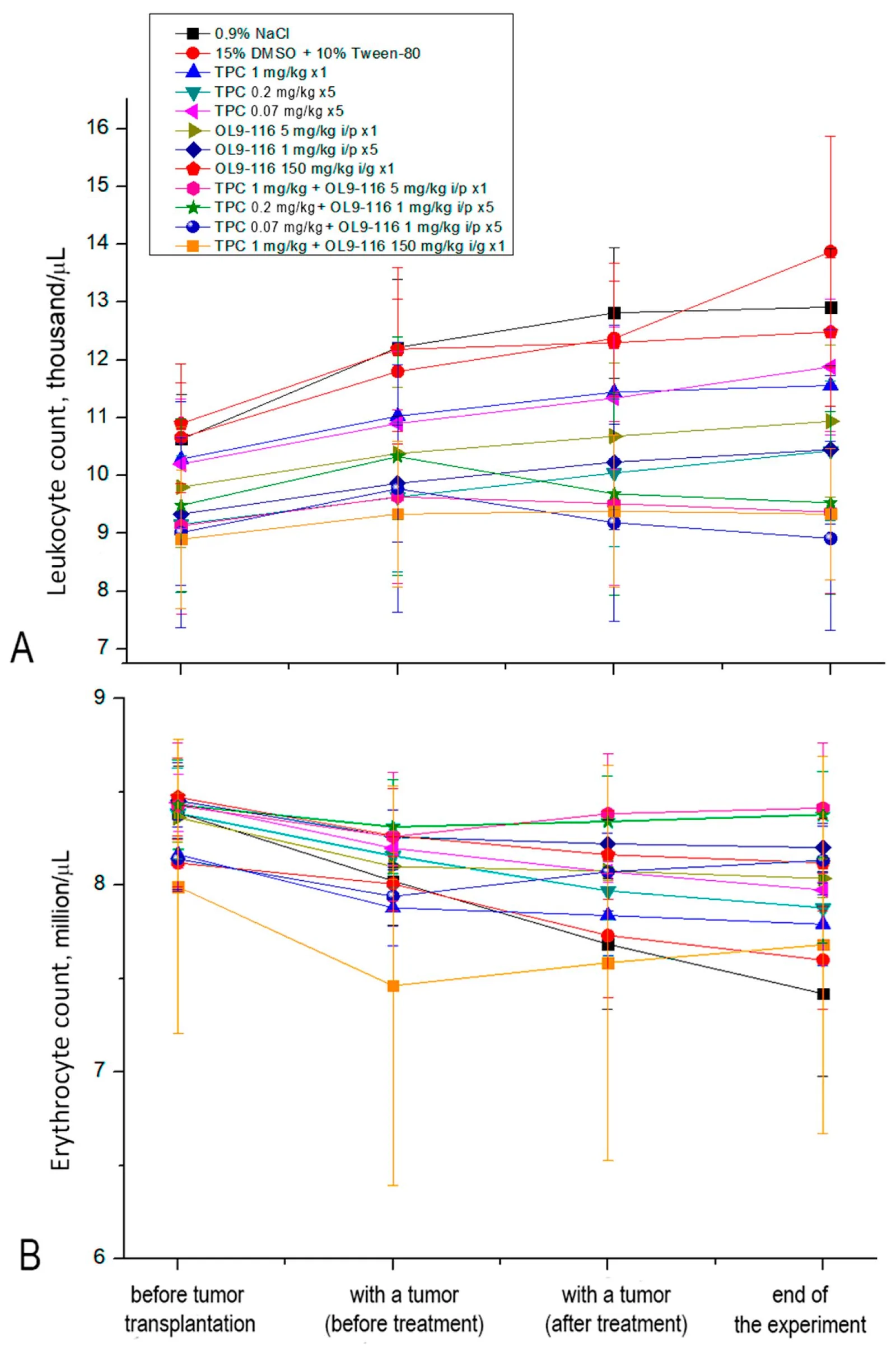
Dynamics of leukocyte (**A**) and erythrocyte (**B**) count in mice bearing the transplanted RShM-5 cervical cancer tumor model. Data are presented as mean ± standard deviation (SD). The number of animals was n = 7 per group.

During the experiment, serial hematologic evaluations were performed before tumor transplantation, prior to the initiation of therapy, on the last day of drug administration, and at the end of the observation period. We conducted an assessment of the number of leukocytes and erythrocytes in the peripheral blood of mice bearing the RShM-5 tumor model. This made it possible to trace the impact of both the tumor process and the different treatment regimens on hematopoiesis. Inter- and intra-group variability of these parameters in clinically healthy animals at baseline fell within the established reference intervals described for laboratory mouse strains, reflecting physiological inter-individual variability rather than pathology. The data are presented in Figure 6 and in Tables S2 and S3.

Throughout the experiment, mice bearing the transplanted tumor exhibited an increase in total leukocyte count, which can be interpreted as a manifestation of tumor-associated systemic inflammation and leukocytosis in tumor-bearing animals [31,32]. At the same time, intergroup differences in leukocyte dynamics presumably reflected the specific features of tumor growth and the impact of the applied therapy on the magnitude of the inflammatory response.

Overall, the temporal changes in leukocyte and erythrocyte concentrations in the 15% DMSO + 10% Tween-80 group were comparable to those observed in the 0.9% NaCl group. In both groups, progressive tumor growth was accompanied by pronounced leukocytosis and a decrease in erythrocyte counts. These findings indicate that the vehicle itself does not have any intrinsic hematoprotective effect on peripheral blood parameters.

Course administration of TPC at single doses of 0.07 and 0.2 mg/kg, as well as a single administration of TPC at 1 mg/kg, did not prevent the development of tumor-associated leukocytosis. In all groups receiving TPC monotherapy, a gradual increase in leukocyte counts relative to baseline was observed; however, the magnitude of this effect was lower than in the control groups. At the same time, the decrease in erythrocyte counts was less pronounced than in the 0.9% NaCl group, indicating partial preservation of the erythroid lineage of hematopoiesis.

OL9-116 was found to have a significant protective effect on peripheral blood parameters. However, its efficacy depended on the dosing regimen. The most prominent improvement in peripheral blood parameters was observed with a 5-day course of OL9-116 at 1 mg/kg and with a single administration at 5 mg/kg. Under these conditions, leukocyte counts remained close to baseline values and erythrocyte levels were virtually unchanged throughout the experiment.

At the same time, the protective effect of a single 150 mg/kg dose of OL9-116 was less pronounced: by the end of the experiment, leukocyte counts had increased to levels exceeding those in the low-dose OL9-116 groups, although they remained below or comparable to control values. In contrast, erythrocyte counts remained close to baseline levels, indicating the preservation of the hematoprotective activity of the compound with respect to the erythroid lineage of hematopoiesis. Thus, the OL9-116 150 mg/kg ×1 regimen was less effective than the 1 mg/kg ×5 and 5 mg/kg ×1 schedules. This suggests that fractionated or low-dose administration of OL9-116 may be advantageous.

The combined administration of TPC and OL9-116 was associated with more favorable hematologic dynamics than TPC monotherapy. In all combination groups, the development of tumor-associated leukocytosis was attenuated and erythrocyte counts were better preserved than in the control group and in groups receiving TPC alone. The blood parameters closest to the norm were observed in the group that received a 5-day course of the combination of TPC at a single dose of 0.2 mg/kg and OL9-116 at 1 mg/kg.

Following the initiation of treatment, there was virtually no increase in leukocyte counts, which remained close to baseline values by the end of the experiment. At the same time, this group exhibited the highest erythrocyte levels of all the treatment regimens evaluated. A 5-day course of the combination of TPC at a single dose of 0.07 mg/kg and OL9-116 at 1 mg/kg likewise produced a significant improvement in hematologic parameters compared with TPC monotherapy at the same dose. Leukocyte counts remained substantially below control values and markedly lower than in the group receiving a 5-day TPC monotherapy regimen at 0.07 mg/kg, whereas erythrocyte levels stayed within the physiological range throughout the observation period. In the group that received the combination of TPC 1 mg/kg plus OL9-116 5 mg/kg once, a moderate increase in leukocyte counts was observed relative to baseline. However, the magnitude of the leukocytic response was considerably lower than in the control groups and in the TPC monotherapy group.

Erythrocyte counts remained stable and exceeded the values recorded in animals treated with TPC alone. The combination of TPC 1 mg/kg and OL9-116 150 mg/kg in a single dose likewise limited the development of leukocytosis and helped maintain higher erythrocyte counts compared with controls. Nevertheless, in terms of its effect on leukocyte and erythrocyte counts, this regimen was somewhat inferior to those involving a 5-day course of OL9-116 at 1 mg/kg, as indicated by higher leukocyte counts at the end of the experiment and greater variability in erythrocyte parameters.

Based on the available data, we cannot distinguish between situations where OL9-116 directly affects hematopoiesis and those where it influences blood parameters by reducing tumor burden. Further research is required to elucidate the precise mechanism of action of OL9-116 on blood parameters.

#### 2.6.3. Effect of OL9-116 on Organ Indices in Mice with RShM-5

**Figure 7:**
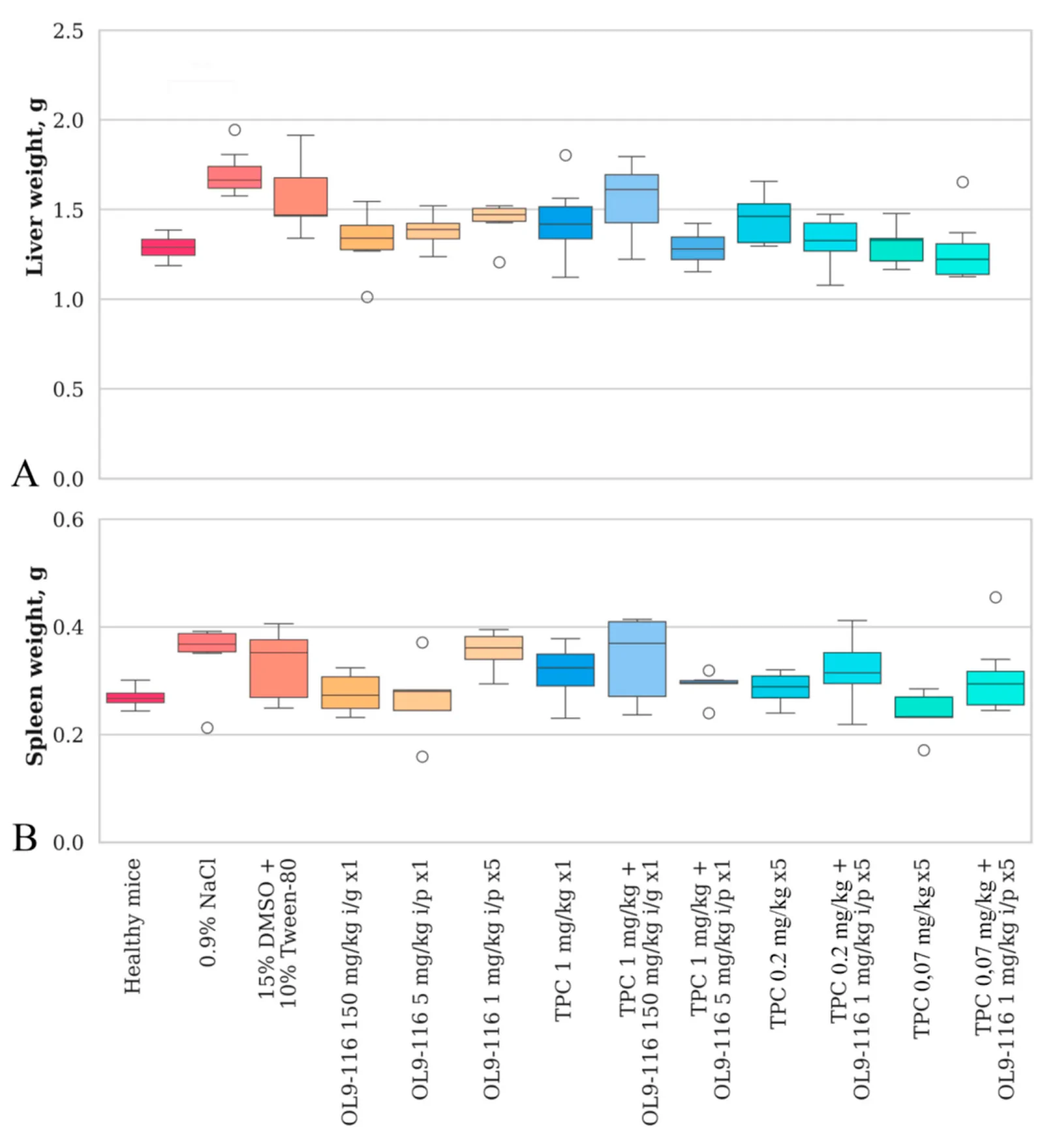
Mean liver weight (**A**) and spleen weight (**B**) in mice bearing the transplanted RShM-5 and in healthy control mice. The number of animals was n = 7 per group. Empty circles (∘) denote outliers.

Analysis of liver and spleen mass in mice bearing the RShM-5 cervical tumor model demonstrated that, across all experimental groups, the parameters under investigation remained within the physiological range and did not exhibit statistically significant shifts relative to either the corresponding vehicle controls or the healthy animal group (Figure 7). Only moderate biological variability in liver and spleen mass was observed in the control cohorts (0.9% NaCl and 15% DMSO + 10% Tween-80). Under administration of TPC, OL9-116, and their combination, the median values of the distributions remained within the same range. There were no signs of hepatomegaly, splenomegaly, or organ atrophy, thereby indicating the absence of pronounced hepato- and splenotoxic effects associated with the therapeutic regimens under investigation.

The obtained data demonstrate a pronounced antitumor effect of OL9-116 in combination with TPC and indicate the possibility of reducing the dose of the main drug, TPC, without loss of efficacy. This opens up the prospect of developing less toxic treatment regimens.

### 2.7. Investigation of the Antitumor Activity of OL9-116 in Combination with Topotecan During Course-Based Combined Administration of the Drugs with Matching Times to Reach Their Maximum Blood Concentrations in a Lewis Lung Carcinoma (LLC) Mouse Model

**Figure 8:**
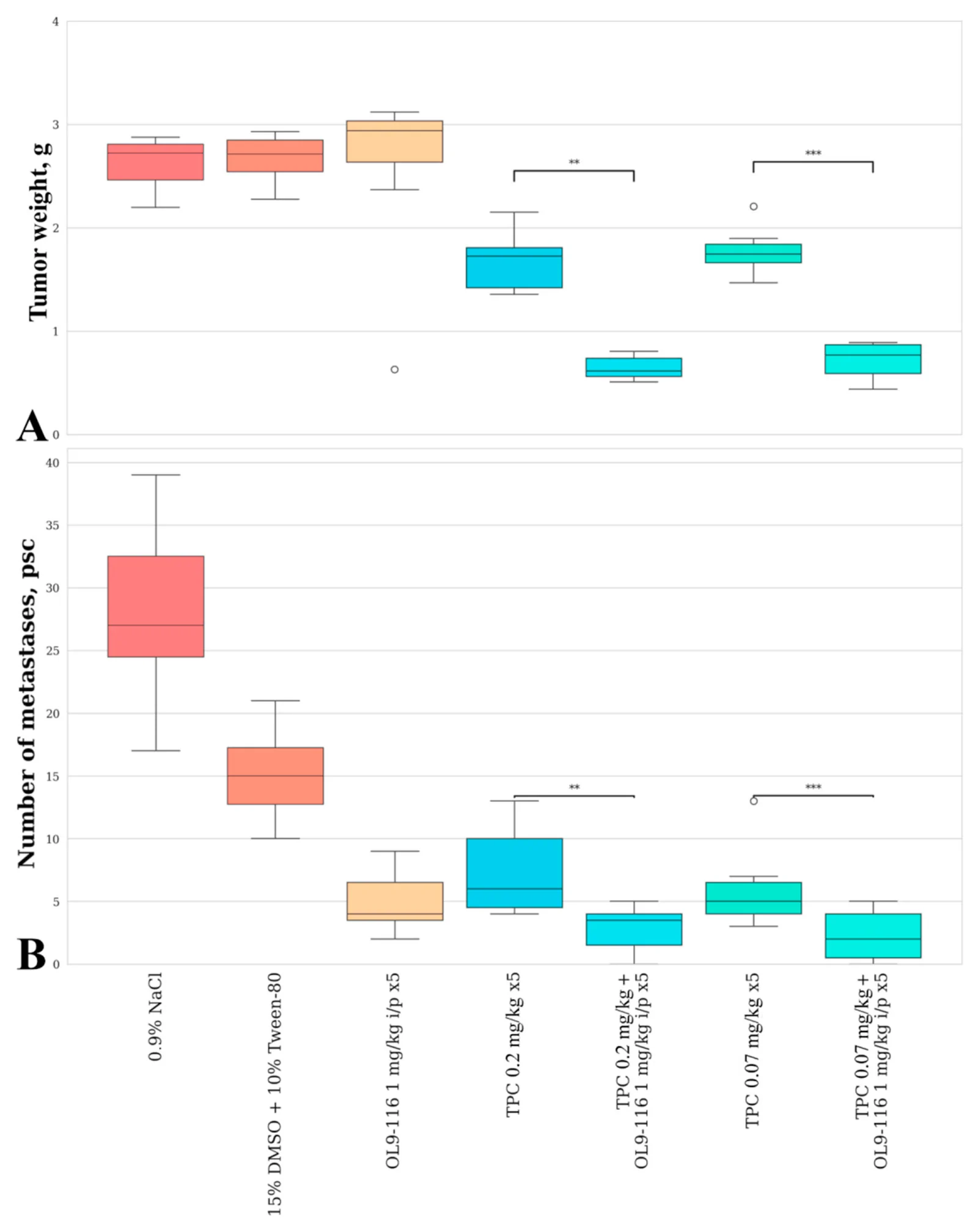
Effect of topotecan (TPC), OL9-116, and their combined course-based administration under pharmacokinetic synchronization on primary tumor nodule mass (**A**) and number of metastases (**B**) in mice bearing Lewis lung carcinoma (LLC). ** *p* < 0.01 and *** *p* < 0.001, Mann–Whitney U test. The number of animals was n = 7 per group. Empty circles (∘) denote outliers.

Based on data obtained at the previous study stage, we evaluated the efficacy of the combination of OL9-116 and TPC under a course-based dosing regimen with synchronized times to reach their maximum blood concentrations in a Lewis lung carcinoma (LLC) tumor model. This model is widely used for the preclinical evaluation of the antitumor and antimetastatic activity of chemotherapeutic agents, including within combination therapy regimens. At the same time, we sought to determine whether the dose of the principal drug, TPC, could be reduced under these conditions without loss of antitumor and antimetastatic efficacy against this tumor model. This appears to be fundamentally important in terms of decreasing systemic toxicity and improving the therapeutic index of the treatment regimen. The experimental results are presented in Figure 8.

#### 2.7.1. Effect of OL9-116 on LLC Tumor Growth and Metastasis

The values of tumor nodule mass were found to be comparable in the physiological control group (0.9% NaCl) and in the vehicle group (15% DMSO + 10% Tween-80) (mean tumor nodule weights of 2.6 and 2.7 g, respectively; no statistically significant differences were observed between these groups). Moreover, the highest number of metastases (28 ± 6) was observed in the 0.9% NaCl group, indicating a significant metastatic process in the absence of any treatment. The number of metastases was lower (16 ± 3) in the 15% DMSO + 10% Tween-80 group than in the 0.9% NaCl group. However, the distributions substantially overlapped, indicating that metastatic activity remained high and that the vehicle itself did not have an antimetastatic effect.

Course intraperitoneal administration of OL9-116 at a dose of 1 mg/kg once daily for 5 days did not result in a change in median tumor nodule mass: its value (2.6 g) was comparable to those in the control and vehicle groups, indicating an absence of an antitumor effect when OL9-116 is used as monotherapy. Nevertheless, this treatment resulted in a distinct reduction in the number of metastases (5 ± 2) compared to the control group (28 ± 6) and the group that received 15% DMSO + 10% Tween-80 (16 ± 3).

Administering TPC at a dose of 0.2 mg/kg once daily for 5 days was accompanied by a 1.7 g reduction in tumor nodule mass (TGI, 37%) compared to the control group, the vehicle group, and mice receiving OL9-116 at 1 mg/kg in a course-based manner. The range of tumor mass values was shifted downwards, indicating a moderate cytostatic/cytotoxic effect of the compound. TPC monotherapy at 0.2 mg/kg suppressed the number of metastases compared with the control groups (7 ± 3). A similar decrease in the number of metastases (6 ± 2) and in primary tumor nodule mass was observed at the lower TPC dose of 0.07 mg/kg (1.8 g; TGI, 33.3%) administered once daily for 5 days, and no pronounced dose-dependent trend was apparent within the tested dose range.

The most significant reduction in tumor nodule mass was observed in the groups receiving 5-day combination therapy with pharmacokinetic alignment of the C_max_ peaks of TPC and OL9-116 for both TPC doses, 0.07 and 0.2 mg/kg (mean tumor nodule weights, 0.7 and 0.6 g; TGI, 74.1% and 77.8%, respectively). The median primary tumor nodule masses in these groups were approximately fourfold lower than in the control and vehicle groups. A statistically significant reduction in tumor mass was produced by combined course therapy with OL9-116 at 1 mg/kg and TPC at a single dose of 0.07 or 0.2 mg/kg compared with course TPC monotherapy at 0.07 or 0.2 mg/kg (** *p* < 0.01 and *** *p* < 0.001). Co-administration of OL9-116 at 1 mg/kg with TPC at 0.07 or 0.2 mg/kg for 5 days likewise resulted in more effective suppression of the number of lung metastases in mice (numbers of metastases, 2.3 ± 1.8 and 2.8 ± 1.6, respectively; ** *p* < 0.01 and * *p* < 0.001 compared with groups receiving TPC alone at doses of 0.07 or 0.2 mg/kg).

#### 2.7.2. Effect of OL9-116 on the State of Peripheral Blood in Mice with LLC

**Figure 9:**
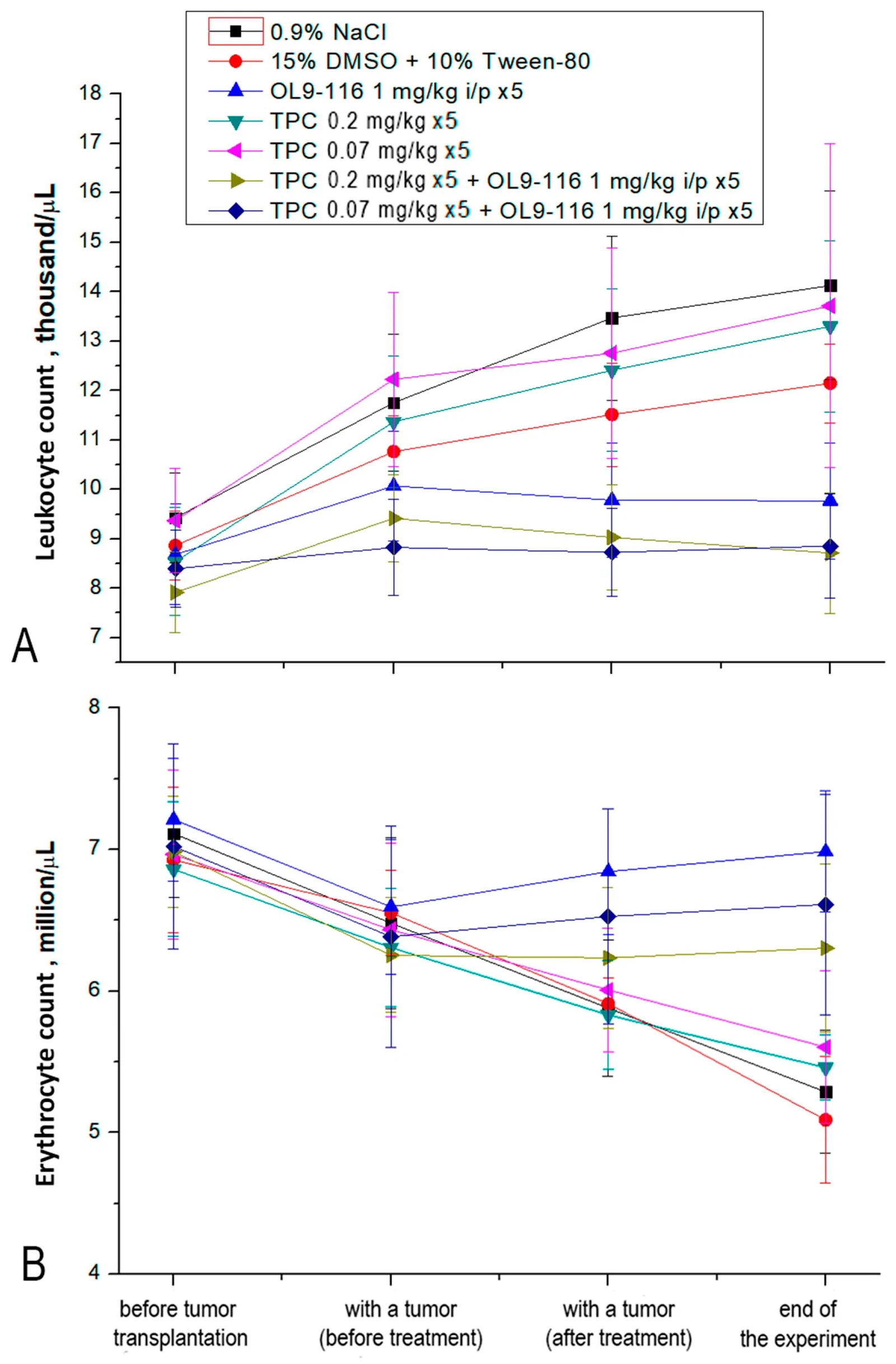
Temporal dynamics of leukocyte (**A**) and erythrocyte (**B**) count in mice bearing a transplanted Lewis lung carcinoma (LLC). Data are presented as mean ± standard deviation (SD). The number of animals was n = 7 per group.

During the experiment, we evaluated the hematological effects of the investigated compounds and their combination in Lewis lung carcinoma-bearing mice over time (prior to tumor cell transplantation, before the initiation of therapy, on the last day of drug administration, and at the end of the experiment). The data are presented in Figure 9 and in Tables S4 and S5.

The temporal dynamics of leukocyte and erythrocyte concentrations in the 15% DMSO + 10% Tween-80 group were virtually indistinguishable from those in the 0.9% NaCl group, indicating an absence of marked intrinsic hematotoxicity of the vehicle under the selected administration regimen.

TPC at both doses, 0.07 and 0.2 mg/kg, exacerbated the leukocytosis inherent to LLC and deepened anemia compared with the 0.9% NaCl control, indicating the presence of dose-independent hematologic toxicity.

OL9-116 exhibited a distinct effect on blood parameters: the leukocyte response was less pronounced, and erythrocyte concentrations returned to baseline values by the end of the study, in contrast to the progressive anemia observed in the control group and in groups receiving TPC monotherapy at 0.07 or 0.2 mg/kg.

Both combinations of OL9-116 at 1 mg/kg with TPC at 0.07 or 0.2 mg/kg showed intermediate or OL9-116-like values: leukocyte counts remained close to the physiological range, and erythrocyte counts were significantly higher than in the control and TPC monotherapy groups. These results indicate that compound OL9-116 potentiates the antitumor activity of TPC and also improves the state of peripheral blood.

#### 2.7.3. Effect of OL9-116 on Organ Indices in Mice with LLC

**Figure 10:**
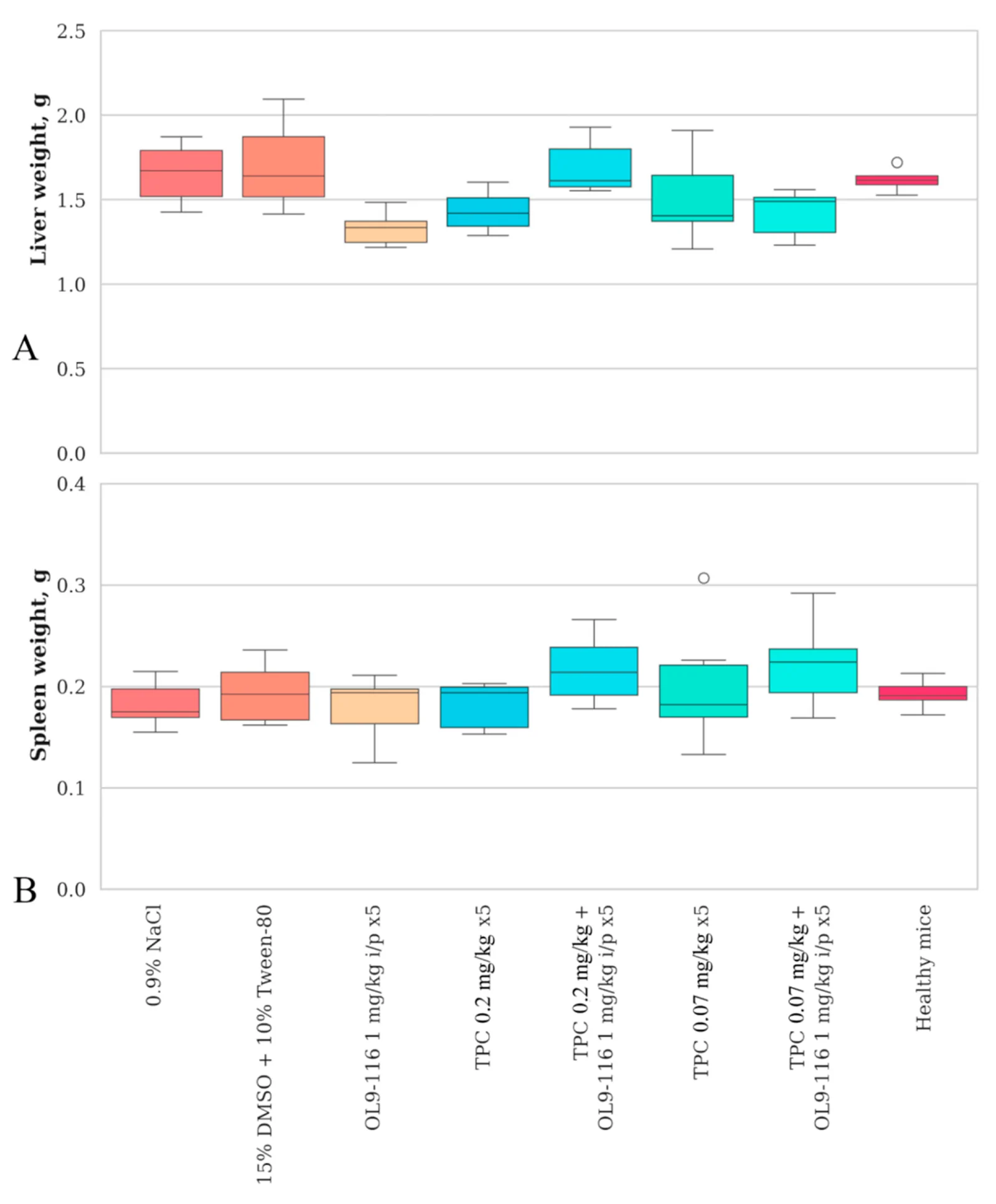
Mean liver (**A**) and spleen (**B**) weight in mice bearing a transplanted Lewis lung carcinoma (LLC) tumor model and in healthy mice. The number of animals was n = 7 per group. Empty circles (∘) denote outliers.

At the end of the experiment, we assessed liver and spleen weights of mice bearing Lewis lung carcinoma (LLC) that were treated with the investigational compounds (Figure 10). Overall, the organ weights of all the treatment groups were comparable to those of healthy animals, indicating an absence of overt hepato- and splenotoxicity at the employed doses and dosing schedules (Figure 10).

In the OL9-116 monotherapy group, which received a once-daily dose of 1 mg/kg for 5 consecutive days, a statistically significant but moderate reduction in liver weight was observed compared with tumor-bearing control mice. However, the absolute values remained within the range of variability characteristic of healthy animals and therefore do not suggest clinically relevant liver injury.

Combined administration of TPC with OL9-116 did not result in any additional increase in liver or spleen weight compared with tumor-bearing control groups and healthy mice. This allows these regimens to be regarded as not being associated with overt toxicity towards parenchymal and lymphoid organs.

### 2.8. The Compound OL9-116 Has No Effect on the Locomotor and Exploratory Behavior of Healthy Mice in Open Field Test

Next we tested the potential adverse effects of OL9-116 on healthy mice in the open field test. This test is a classical method for assessing the behavior of laboratory animals, most commonly mice and rats. We measured three parameters—distance travelled, time spent in the center, and number of rearings—for three groups of mice (DMSO, 5 mg/kg and 50 mg/kg OL9-116). To assess the acute and delayed effects of OL9-116, open-field parameters were analyzed using a two-way repeated-measures ANOVA, with treatment as the between-subject factor and time (2 h and 3 weeks) as the within-subject factor.

**Figure 11:**
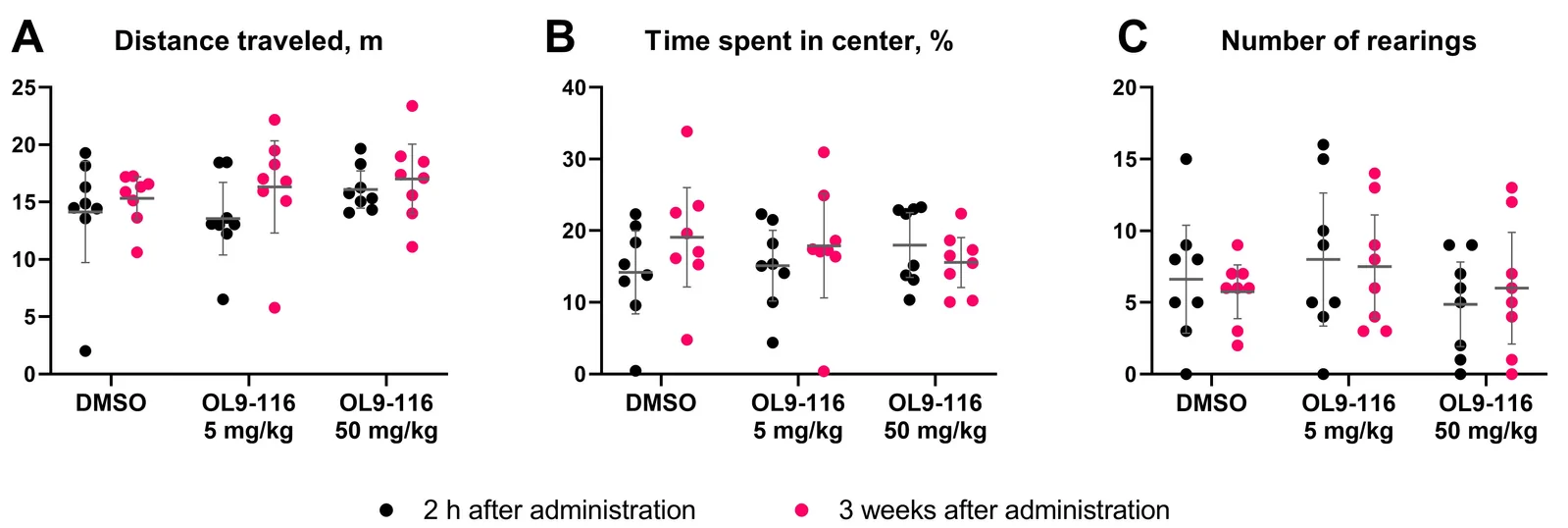
Effects of OL9-116 in open field test 2 h (2 h) and 3 weeks after administration. (**A**) Distance traveled, (**B**) percentage of time spent in the center of the arena, and (**C**) number of rearings in mice treated with vehicle (DMSO) or OL9-116 at two doses, 5 and 50 mg/kg. The same animals were tested at both time points. Individual values are shown; horizontal lines and error bars represent the mean and 95% confidence interval, respectively (n = 8 per group). Data were analyzed using a two-way repeated-measures ANOVA, with treatment as the between-subject factor and time as the within-subject factor, followed by Šídák-adjusted multiple comparisons.

Two-way repeated-measures ANOVA revealed no significant effect of treatment with OL9-116 at either dose, 5 or 50 mg/kg, compared with the control group (DMSO) on distance travelled, F_2,21_ = 0.733, *p* = 0.493, no treatment × time interaction, F_2,21_ = 0.543, *p* = 0.589, and only a borderline overall effect of time, F_1,21_ = 4.258, *p* = 0.052 (Figure 11A). Time spent in the center was not affected by treatment, F_2,21_ = 0.005, *p* = 0.995, time, F_1,21_ = 1.458, *p* = 0.241, or their interaction, F_2,21_ = 2.237, *p* = 0.132 (Figure 11B). Similarly, the number of rearings showed no significant treatment effect, F_2,21_ = 1.312, *p* = 0.290, time effect, F_1,21_ = 0.004, *p* = 0.948, or treatment × time interaction, F_2,21_ = 0.234, *p* = 0.794 (Figure 11C). Šídák-adjusted post hoc comparisons detected no significant differences between the DMSO and OL9-116 groups at either 2 h or 3 weeks and no significant changes between the two time points within any treatment group. Thus, under the present experimental conditions, OL9-116 produced no detectable acute or delayed effects on the assessed locomotor and exploratory parameters.

## 3. Materials and Methods

### 3.1. Chemicals and Reagents

The usnic acid derivative OL9-116, a TDP1 inhibitor, was synthesized as described previously [33]. Topotecan was obtained from USP (Hyderabad, India); methanol and acetonitrile were of chromatographic grade (Chimmed, Moscow, Russia); dimethyl sulfoxide (DMSO) was purchased from Sigma (St. Louis, MO, USA); formic acid and Tween-80 were obtained from Panreac (Barcelona, Spain). Water was purified using a Direct-Q3 UV system (Millipore S.A.S., Molsheim, France). ZnSO_4_ was purchased from Merck (Darmstadt, Germany); 0.9% saline (NaCl) was obtained from Renewal (Novosibirsk, Russia); HistoSafe^®^, 10% buffered formalin, was purchased from BioVitrum (Novosibirsk, Russia).

### 3.2. Assessment of the Carcinogenic Safety of OL9-116 by Analyzing Its Mutagenic Properties Using a Modified Ames Assay In Vitro

The Ames assay was performed using the plate-incorporation method of exposure in accordance with ISO Standard 10993-3:2003 [34] based on the work of Maron and Ames [35]. Compounds were tested for mutagenicity using *Salmonella typhimurium* tester strains TA100 and TA98 both with and without the rat-liver S9 preparation. Prior to sacrifice, male Wistar rats were pretreated with a mixture of polychlorinated biphenyls (Sovol, Orgsteklo Production Association, Dzerzhinsk, Russia) to induce CYP enzymes [36]. The components of the S9 mix were dissolved in 0.1 M phosphate buffer, pH 7.4 (Reachem, Moscow, Russia), 33 mM potassium chloride (Reachem, Moscow, Russia), 8 mM magnesium chloride (Reachem, Moscow, Russia), 5 mM glucose 6-phosphate (Reanal, Budapest, Hungary), 4 mM nicotinamide adenine dinucleotide phosphate (NADP, Sigma, Ronkonkoma, NY, USA) and S9 homogenate at a concentration of 0.1 mL/mL of the mix (10%). The chemicals tested were combined with the *Salmonella typhimurium* tester strain and the metabolic activation mixture (as required). The contents were then mixed with molten agar and poured onto glucose minimal-medium agar plates. Plates were incubated at 37 °C for 72 h. After the incubation, the number of revertant colonies on each plate was scored. All experiments were performed twice, with 3 technical replicates in each experiment.

As positive controls for frame-shift activity with the TA98 strain in the absence of metabolic activation, we used 2,7-diamino-4,9-dihydroxy-5,10-dioxo-4,5,9,10-tetrahydro-4,9-diazopyrene (DDDTDP, Reachem, Moscow, Russia) at a dose of 6.7 × 10^−2^ mol/plate; in the presence of S9 extract, we used 2-aminofluorene (2-AF, CAS#153-78-6, Sigma, Ronkonkoma, NY, USA) at a dose of 1.1 × 10^−1^ mol/plate and benz(a)pyrene (BaP, CAS#50-32-8, Fluka, Seelze, Germany) at a dose of 4 × 10^−2^ mol/plate. As positive controls for base-substitution activity with TA100 in the absence of S9 fraction, we used sodium azide (NaN_3_, CAS# 26628-22-8, Sigma, Ronkonkoma, NY, USA) at a dose of 6.8 × 10^−2^ mol/plate, and in the presence of metabolic activation, we used 2-AF at a dose of 1.1 × 10^−1^ mol/plate and benz(a)pyrene at a dose of 4 × 10^−2^ mol/plate.

For the test compound OL9-116, the following doses were used: 4.4 × 10^−9^, 4.4 × 10^−8^, 4.4 × 10^−7^ mol/plate. The 0.4% DMSO concentration was not exceeded in any tested concentration.

### 3.3. Assessment of OL9-116 Epigenetic Activity Using the HeLa TI Cell-Based Assay

HeLa TI cells were cultured in Dulbecco’s Modified Eagle’s Medium (DMEM; Paneco, Moscow, Russia) containing 4.5 g/L glucose and supplemented with 10% (*v*/*v*) heat-inactivated fetal bovine serum (Biosera, Cholet, France), penicillin (50 U/mL), streptomycin (50 μg/mL) (Paneco, Moscow, Russia), and 2 mM L-glutamine (Paneco, Moscow, Russia). Cells were maintained at 37 °C in a humidified atmosphere containing 5% CO_2_. The cell line was obtained from the biocollection of the Blokhin National Medical Research Center. HeLa TI cells were seeded into 24-well plates at a density of 2 × 10^4^ cells per well. Following cell attachment, the cells were treated with OL9-116 at concentrations of 10 and 20 µM. Trichostatin A (TSA), a histone deacetylase inhibitor, was used as a positive control at 0.25 μM. The final concentration of DMSO in the culture medium did not exceed 0.4%. Untreated HeLa cells and DMSO-treated cells were used as negative controls. After 24 h of treatment, the medium was replaced with fresh drug-free medium, and the cells were cultured for an additional 48 h. The cells were then harvested, washed with PBS, and analyzed by flow cytometry on a FACSCanto II flow cytometer (BD Biosciences, San Jose, CA, USA) as previously described []. Flow cytometry data were analyzed using one-way analysis of variance (one-way ANOVA) followed by Dunnett’s multiple comparisons test. Differences were considered statistically significant at *p* < 0.05. The experiment was repeated three times, with two technical replicates for each.

### 3.4. Cytokinesis-Block Micronucleus (CBMN) Assay

The cytokinesis-block micronucleus (CBMN) assay was performed in accordance with the OECD Guideline for the Testing of Chemicals No. 487 (OECD TG 487) for the in vitro mammalian cell micronucleus test [19]. This test is a genotoxicity assay for the detection of micronuclei in the cytoplasm of interphase cells; micronuclei may originate from acentric chromosome fragments or from whole chromosomes that fail to migrate to the poles during anaphase, thus allowing for the detection of both clastogenic and aneugenic activity of test substances. This test is widely used in genotoxicological and molecular epidemiological studies to assess chromosomal damage, genomic instability, and individual sensitivity to physical and chemical genotoxic agents, as well as a biomarker of cancer risk [37,38,39,40,41].

The genotoxic and cytotoxic potential of compound OL9-116 was evaluated using the cytokinesis-block micronucleus (CBMN) assay in human peripheral blood lymphocytes. Venous blood was collected from two healthy donors (one male and one female, aged 20–30 years, with no harmful habits) into heparinized tubes and stored at 4 °C for 24 h prior to cultivation. Subsequently, 200 µL of whole blood was added to culture flasks containing 3.8 mL of RPMI-1640 medium (Paneco, Moscow, Russia). T-lymphocyte proliferation was stimulated with phytohemagglutinin (PHA, Paneco, Moscow, Russia) at a final concentration of 8 µg/mL. Cultures were incubated at 37 °C in a 5% CO_2_ atmosphere for 44 h.

After this period, OL9-116 was added to experimental cultures at final concentrations of 0.1, 1, 10, and 100 µM (per 1 mL of culture). Ifosfamide (IFO, an alkylating agent) and decitabine (Dac, a DNA methyltransferase inhibitor) were used as positive controls, while DMSO at a final concentration of 1% served as the negative control. All compounds were added simultaneously. Following a 24 h exposure to the test compounds, cytochalasin B was added to all cultures at a final concentration of 6 µg/mL to block cytokinesis, and incubation was continued for an additional 28 h under the same conditions. At the end of the total culture period (96 h after blood addition), cells were harvested by centrifugation, fixed with a chilled mixture of glacial acetic acid and methanol (1:3, *v*/*v*), and stained with Giemsa solution. Slides were coded and scored blindly under a light microscope. The analysis was performed in two stages. In the first stage, 500 randomly selected cells (regardless of the number of nuclei) were examined to determine the number of cells with one, two, three, four, or more nuclei, as well as cells in mitosis and apoptosis. Based on these data, the cytokinesis-block proliferation index (CBPI), reflecting the average number of nuclei per cell, was calculated as the ratio of total nuclei to the number of cells scored (N = 500).

In the second stage, 2000 binucleated cells (with two clearly distinguishable main nuclei) were evaluated for the frequency of micronuclei (MN), nucleoplasmic bridges (NPBs), and nuclear buds (NBUDs). Micronuclei were scored as small round structures with a diameter between 1/16 and 1/3 of the main nucleus, not overlapping or connected to it. Nucleoplasmic bridges were identified as continuous chromatin strands linking the two main nuclei. Nuclear buds were defined as protrusions of the nuclear membrane containing chromatin and connected to the nucleus by a thin stalk. Statistical significance of differences between groups was assessed using Pearson’s chi-square test (with significance levels set at *p* < 0.05 and *p* < 0.01). Two technical replicates were performed for each donor.

### 3.5. Laboratory Animals

Animals were obtained from the breeding colony of the Institute of Chemical Biology and Fundamental Medicine, Siberian Branch of the Russian Academy of Sciences (ICBFM SB RAS, Novosibirsk, Russia). The experiments were performed on sexually mature 5–6-month-old male mice of the C57BL/6J (C57BL) and CBA/Lac strains. Animals were housed on wood shavings in plastic cages with free access to food and water. All animal experiments described in this study were conducted in accordance with international guidelines for the care and use of laboratory animals [Directive 2010/63/EU of the European Parliament and of the Council of 22 September 2010 on the protection of animals used for scientific purposes] and protocols approved by the Inter-Institutional Bioethics Committee of the Institute of Cytology and Genetics SB RAS, approval No. 21.11 dated 30 May 2014.

The investigation was carried out on CBA inbred male mice 16–20 weeks old weighing 37.1 ± 3.6 g at the Center for Genetic Resources of Laboratory Animals of the Institute of Cytology and Genetics, Siberian Branch of the Russian Academy of Sciences. The animals were kept under the standard conditions of the vivarium, which included artificial 14 h lighting, 60% humidity, a temperature of 23 °C and free access to balanced food and water. All the procedures involving experimental animals were performed in accordance with the international rules for the treatment of animals (Directive 2010/63/EU) and the Order of the Ministry of Health of the Russian Federation on Approval of the Rules of Good Laboratory Practice of 04/01/2016 No. 199n (registered on 15 August 2016 No. 43232).

### 3.6. Tumor Strains

The following transplantable tumor strains were used in this study: Lewis lung carcinoma (hereafter LLC), a rapidly growing lung tumor that metastasizes to the lungs and exhibits selective sensitivity to antitumor agents; this strain was obtained from the cell repository of the Institute of Cytology and Genetics (Novosibirsk, Russia) and is maintained in C57BL/6 mice. Cervical cancer RShM-5, a highly differentiated tumor characterized by slow in vivo growth; this strain was obtained from the bank of deep-frozen cell cultures of the N.N. Blokhin National Medical Research Center of Oncology, Ministry of Health of the Russian Federation, and is maintained in CBA/Lac mice [42]. In the in vivo experiments, second-passage tumors of these strains were used.

For transplantation of LLC, the original tumor tissue was homogenized in Hank’s medium (Paneco, Moscow, Russia). The resulting cell suspension (0.2 mL, 4 × 10^5^ cells per mouse) was injected intramuscularly into the thigh region of the right hind limb, where a primary tumor nodule subsequently developed.

For modeling RShM-5, tumor tissue was minced to a homogeneous consistency, Hank’s medium (Paneco, Moscow, Russia) was added, and 0.5 mL of the resulting suspension (2.5 × 10^6^ cells per mouse) was injected subcutaneously into the right axillary region, where a primary tumor nodule subsequently developed.

### 3.7. Preparation of Test Compounds

For in vitro experiments, OL9-116 was dissolved in 100% dimethyl sulfoxide (DMSO).

For in vivo experiments, OL9-116 was first dissolved in dimethyl sulfoxide (DMSO) (final concentration in solution 15%), then Tween-80 was added (final concentration 10%) and the mixture was thoroughly vortexed on an orbital shaker. Physiological saline (0.9% NaCl) was then added and the suspension was shaken again. The resulting suspension was administered either intragastrically or intraperitoneally in a volume of 0.2 mL. Topotecan (TPC) was administered intraperitoneally in 0.9% NaCl solution in a volume of 0.2 mL.

### 3.8. Extrapolation of the Clinically Used Course Regimen of Topotecan Administration to In Vivo Therapy of the RShM-5 Tumor Model in Mice Using an Interspecies Dose Conversion Factor

In clinical practice, topotecan is administered in 5-day courses at doses ranging from 0.5 to 1.5 mg/m^2^. We selected an intermediate single dose of 1 mg/m^2^ and converted it using an interspecies dose-translation formula [43]:

S = (k × M^0.67^)/100,

where S is the body surface area, k is the species-specific coefficient (12 for mice), and M is the body weight in kg (in this experiment, 0.03 ± 0.001 kg). Thus, the body surface area of the mice in the experiment is 0.012 m^2^, and the single dose for a mouse is 0.012 m^2^ × 1 mg/m^2^ = 0.012 mg/mouse, corresponding to 0.4 mg/kg.

Treatment was initiated 48 h after transplantation of tumor cells. TPC was administered twice on days 3 and 7, or five times daily from day 3 to day 7. The animals were sacrificed on day 21. Antitumor activity of the drugs was evaluated at the end of the experiment based on the weight of the primary tumor nodule, and toxic effects were assessed by liver and spleen weights. In addition, for each treatment group, the TGI value was calculated according to the following formula:

TGI, % = (M_control_ − M_treated_)/M_control_ × 100,

where M_control_ is the mean tumor weight in the control group and M_treated_ is the mean tumor weight in the groups receiving the study drugs.

Data obtained in animal experiments were subjected to statistical analysis using the nonparametric Mann–Whitney U test with Bonferroni correction. The results were considered statistically significant at *p* < 0.05. The number of animals in each comparison group was n = 7.

### 3.9. Pharmacokinetic Study of OL9-116 in the Blood of Healthy Mice Following Intraperitoneal Administration

Pharmacokinetic evaluation of OL9-116 following intraperitoneal administration at doses of 150 and 5 mg/kg was performed using a previously developed and validated LC–MS/MS assay [17]. Briefly, at 0.5, 1.0, 1.5, 2.0, 2.5, 3.0, 4.0, 5.0, 6.0, 7.0, 8.0, 9.0, 10.0, and 24.0 h after dosing, 10 µL blood aliquots were collected from the tail vein. Each aliquot was mixed with 10 µL of aqueous 0.2 M ZnSO_4_, vortex-mixed, and incubated at room temperature for 15–20 min. Subsequently, 100 µL of acetonitrile containing the internal standard was added, and the samples were vortexed for 20–30 s and centrifuged for 10 min at 13,400 rpm (MiniSpin, Eppendorf, Hamburg, Germany). The supernatant was transferred to an autosampler vial and analyzed by high-performance liquid chromatography (HPLC) with tandem mass-spectrometric detection in multiple-reaction monitoring (MRM) mode. Chromatographic separation was performed in reversed-phase mode on a C8 column, using water and methanol containing 0.1% formic acid as mobile phases. Pharmacokinetic parameters were calculated using PKSolver 2.0 (China Pharmaceutical University, Nanjing, China) [44] and are presented as mean values ± standard deviation.

A relative bioavailability comparison was conducted between the two drug formulations by calculating the ratio of the two values of AUC and dose ratio via the following formula [45]:

F = (AUCi.p./AUCi.g.) × (Di.g./Di.p.),

where AUCi.p. and AUCi.g. are the values of the area under the concentration–time curve obtained in an experiment of intraperitoneal and intragastric drug administration, respectively, and Di.g. and Di.p. are the doses in intragastric and intraperitoneal drug administration.

### 3.10. Investigation of the Antitumor Activity of OL9-116 in Combination with Topotecan in the RShM-5 Tumor Model

Animals were randomly assigned to twelve groups (n = 7 per group). Treatment was initiated 48 h after tumor cell transplantation, and all animals in each group received the respective substances according to the following dosing regimens:

• Control group: mice received an intraperitoneal injection of 0.9% NaCl (saline) at a volume of 200 µL;
• 15% DMSO + 10% Tween-80: mice were administered intraperitoneally a solution containing 15% DMSO and 10% Tween-80 in 0.9% NaCl in a volume of 200 µL;
• OL9-116 1 mg/kg × 5 i/p: mice received once-daily intraperitoneal injections of OL9-116 for 5 consecutive days at a dose of 1 mg/kg per injection (total cumulative dose 5 mg/kg); OL9-116 was dissolved in a vehicle comprising 15% DMSO, 10% Tween-80, and 0.9% NaCl, and was administered in a volume of 200 µL;
• OL9-116 5 mg/kg × 1 i/p: mice received a single intraperitoneal injection of OL9-116 at a dose of 5 mg/kg;
• OL9-116 150 mg/kg × 1 i/g: mice received a single intragastric administration (oral gavage) of OL9-116 at a dose of 150 mg/kg;
• TPC 1 mg/kg × 1 i/p: mice received a single intraperitoneal injection of TPC at a dose of 1 mg/kg;
• OL9-116 5 mg/kg × 1 i/p + after 2 h TPC 1 mg/kg × 1 i/p: mice received a single intraperitoneal injection of OL9-116 at a dose of 5 mg/kg, followed 2 h later by a single intraperitoneal injection of TPC at a dose of 1 mg/kg;
• OL9-116 150 mg/kg × 1 i/g + after 3 h TPC 1 mg/kg × 1 i/p: mice received a single intragastric administration of OL9-116 at a dose of 150 mg/kg, followed 3 h later by a single intraperitoneal injection of TPC at a dose of 1 mg/kg;
• TPC 0.2 mg/kg × 5 i/p: mice received once-daily intraperitoneal injections of TPC at a dose of 0.2 mg/kg for 5 consecutive days.;
• OL9-116 1 mg/kg × 1 i/p + after 2 h TPC 0.2 mg/kg × 5 i/p: mice received intraperitoneal injections of OL9-116 at a dose of 1 mg/kg once daily for 5 consecutive days, and, 2 h after each OL9-116 administration, intraperitoneal injections of TPC at a dose of 0.2 mg/kg, also once daily for 5 consecutive days;
• TPC 0.07 mg/kg × 5 i/p: mice received once-daily intraperitoneal injections of TPC at a dose of 0.07 mg/kg for 5 consecutive days;
• OL9-116 1 mg/kg × 1 i/p + after 2 h TPC 0.07 mg/kg × 5 i/p: mice received intraperitoneal injections of OL9-116 at a dose of 1 mg/kg once daily for 5 consecutive days, and, 2 h after each OL9-116 administration, intraperitoneal injections of TPC at a dose of 0.07 mg/kg, once daily for 5 consecutive days.

The experiment was terminated 21 days after transplantation of RShM-5 tumor cells. The antitumor effects of the test compounds were evaluated based on the weight of solid RShM-5 tumors. To assess the toxic effects of the investigated agents, serial hematological analysis of peripheral blood was performed throughout the study (before tumor cell transplantation, prior to treatment initiation, after drug administration, and at the end of the experiment). Hematological measurements were carried out using a Swelab Alfa automated hematology analyzer (Boule Diagnostics AB, Spånga, Sweden). Red blood cell and white blood cell counts were determined, and liver and spleen weights were measured at the end of the experiment as additional indicators of toxicity. For each treatment group, the TGI was calculated. Data obtained in animal experiments were subjected to statistical analysis using the non-parametric Mann–Whitney U test to perform pairwise comparisons between groups receiving different topotecan regimens and the corresponding groups treated with topotecan plus OL9-116. No correction for multiple comparisons was performed. The results were considered statistically significant at *p* < 0.05. The number of animals in each comparison group was n = 7.

### 3.11. Investigation of the Antitumor Activity of OL9-116 in Combination with Topotecan in a Lewis Lung Carcinoma (LLC) Model

Animals were randomly allocated to seven groups (n = 7 per group). Treatment was initiated 48 h after transplantation of tumor cells; animals in all groups received the investigational compounds according to the following treatment regimens:

• Control group: mice received an intraperitoneal injection of 0.9% NaCl (saline) at a volume of 200 µL;
• 15% DMSO + 10% Tween-80: mice were administered intraperitoneally a solution containing 15% DMSO and 10% Tween-80 in 0.9% NaCl in a volume of 200 µL;
• OL9-116 1 mg/kg × 5 i/p: mice received once-daily intraperitoneal injections of OL9-116 for 5 consecutive days at a dose of 1 mg/kg per injection (total cumulative dose 5 mg/kg); OL9-116 was dissolved in a vehicle comprising 15% DMSO, 10% Tween-80, and 0.9% NaCl, and was administered in a volume of 200 µL;
• TPC 0.2 mg/kg × 5 i/p: mice received once-daily intraperitoneal injections of TPC at a dose of 0.2 mg/kg for 5 consecutive days;
• OL9-116 1 mg/kg × 1 i/p + after 2 h TPC 0.2 mg/kg × 5 i/p: mice received intraperitoneal injections of OL9-116 at a dose of 1 mg/kg once daily for 5 consecutive days, and, 2 h after each OL9-116 administration, intraperitoneal injections of TPC at a dose of 0.2 mg/kg, also once daily for 5 consecutive days;
• TPC 0.07 mg/kg × 5 i/p: mice received once-daily intraperitoneal injections of TPC at a dose of 0.07 mg/kg for 5 consecutive days;
• OL9-116 1 mg/kg × 1 i/p + after 2 h TPC 0.07 mg/kg × 5 i/p: mice received intraperitoneal injections of OL9-116 at a dose of 1 mg/kg once daily for 5 consecutive days, and, 2 h after each OL9-116 administration, intraperitoneal injections of TPC at a dose of 0.07 mg/kg, once daily for 5 consecutive days.

Animals were removed from the experiment on the 21st day after transplantation of tumor cells. The antitumor efficacy of the treatments was evaluated based on the weight of solid LLC tumors and the number of lung metastases. Lung metastases were counted microscopically (biological research microscope MBI-3, LOMO, St. Petersburg, Russia) at threefold magnification after fixation in 10% formalin. The toxic effects of the investigational compounds were assessed by dynamic hematological analysis of peripheral blood at key time points in the experiment (before tumor cell transplantation, before initiation of therapy, on the last day of drug administration, and at the end of the experiment). Blood analysis was performed using a Swelab Alfa automated hematology analyzer (Boule Diagnostics AB, Sweden); in addition, liver and spleen weights were determined at the end of the experiment to characterize toxicity. For each treatment group, TGI was also calculated. Data obtained from the animal experiments were subjected to statistical evaluation using the nonparametric Mann–Whitney U test to perform pairwise comparisons between groups receiving different topotecan regimens and the corresponding groups treated with topotecan plus OL9-116. No correction for multiple comparisons was performed. The results were considered statistically significant at *p* < 0.05. The number of animals in each comparison group was n = 7.

### 3.12. Studying the Effect of the Compound OL9-116 on the Locomotor and Exploratory Behavior of Healthy Mice in Open Field Test

Animals were randomly allocated to three groups (n = 8 per group): treatment with DMSO as a vehicle; OL9-116 5 mg/kg and 50 mg/kg. All the tests were conducted 2 h and three weeks after treatment. The open field test was conducted using a circular arena (40 cm in diameter) surrounded by a white plastic wall (25 cm in height) and illuminated from below through a matte semi-transparent floor by two 12 W halogen lamps positioned 40 cm beneath the arena floor. Each mouse was placed near the wall, and its behavior was recorded for 5 min using a digital video camera (Sony, Tokyo, Japan) located 80 cm from the arena. The arena was cleaned after each test session. Video footage was analyzed frame by frame using custom EthoStudio software v 2.0. Horizontal locomotor activity (path length) and time spent in the center of arena were automatically measured, while vertical locomotor activity (rearings) was measured manually. Data are presented as means with 95% confidence intervals. The effects of treatment and time were analyzed using a two-way repeated-measures ANOVA, with treatment as a between-subject factor and time as a within-subject factor. RM-ANOVA results are reported as F(df1, df2) with the corresponding *p*-value. The F-statistic quantifies the strength of the tested effect relative to unexplained variability, while df1 and df2 indicate the corresponding degrees of freedom.

Model residuals were assessed for approximate normality using the Shapiro-Wilk test and normal Q-Q plots. Šídák-adjusted multiple comparisons were performed where appropriate. Differences were considered statistically significant at *p* < 0.05.

## 4. Conclusions

In the present study, we performed a comprehensive evaluation of the usnic acid derivative OL9-116, a TDP1 inhibitor, in terms of safety and antitumor efficacy when used as an auxiliary agent in topotecan-based regimens in murine solid tumor models RShM-5 and LLC. The results obtained demonstrate a favorable genotoxic and epigenetic safety profile of the compound, optimal pharmacokinetic characteristics following intraperitoneal administration, and a pronounced antitumor and antimetastatic effect in combination with topotecan.

The modified Ames assay performed in *Salmonella typhimurium* strains TA98 and TA100, in the presence and absence of a metabolically active liver S9 fraction, did not reveal any mutagenic activity of OL9-116 across a broad concentration range, indicating that the compound lacks the potential to induce reverse mutations via base-pair substitution or frameshift mechanisms.

The cytokinesis-block micronucleus assay performed in human peripheral blood lymphocytes demonstrated that, at concentrations of 0.1–100 µM, OL9 116 does not cause a statistically significant increase in the frequency of micronuclei, nucleoplasmic bridges or nuclear buds, and does not alter the cytokinesis-block proliferation index (CBPI) compared with the control, indicating the absence of clastogenic and aneugenic effects in this test system.

The lack of reactivation of the epigenetically repressed GFP gene in HeLa TI cells upon treatment with OL9 116, in contrast to the TSA control, further indicates an absence of pronounced epigenetic effects associated with disruption of epigenetic silencing mechanisms under the conditions used.

The pharmacokinetic study demonstrated that intraperitoneal administration of OL9-116 provides substantially higher bioavailability compared with intragastric administration, allowing comparable maximum blood concentrations to be achieved with a thirty-fold lower dose (5 mg/kg i/p versus 150 mg/kg i/g). Comparable C_max_ values and areas under the concentration–time curve (AUC) at the reduced intraperitoneal dose justifies the selection of this route of OL9-116 administration for subsequent in vivo experiments and for its use in combination regimens with topotecan.

In addition, the course topotecan dosing schedule used in clinical practice was extrapolated to the murine RShM-5 cervical carcinoma model using interspecies dose-conversion coefficients, which enabled the selection of a regimen of topotecan at a single daily dose of 0.2 mg/kg for 5 consecutive days that produced marked inhibition of tumor growth with acceptable tolerability for further in vivo studies.

In the RShM-5 cervical cancer model, OL9-116 monotherapy was associated with moderate inhibition of tumor growth, whereas course administration of topotecan at low once-daily doses of 0.07 and 0.2 mg/kg for 5 days produced a more pronounced antitumor effect than a single 1 mg/kg dose, consistent with the well-known schedule dependence of topotecan efficacy. The most marked suppression of tumor growth was achieved with combination therapy, in which OL9-116 was administered intraperitoneally with synchronization of its time to maximum blood concentration with that of topotecan: the TGI value reached 91% at topotecan doses of 0.07 and 0.2 mg/kg in the presence of OL9-116 at 1 mg/kg given for 5 days, which was significantly superior to topotecan monotherapy at a comparable cumulative dose. Liver and spleen weights, as well as hematological parameters, remained within physiological limits, indicating that the addition of OL9-116 to topotecan did not result in a significant increase in toxicity for the evaluated criteria.

In the Lewis lung carcinoma (LLC) model, OL9-116 administered as monotherapy did not exert a significant antitumor effect on the mass of the primary tumor nodule, but promoted a marked reduction in the number of lung metastases compared with the control, indicating its potential as an antimetastatic agent. Five-day course combination therapy with OL9-116 at 1 mg/kg and topotecan at 0.07 or 0.2 mg/kg under pharmacokinetic synchronization led to more than a twofold decrease in primary tumor mass (TGI 74–78%) and a significant reduction in metastatic burden compared with the corresponding topotecan monotherapy regimens. At the same time, OL9-116 exhibited a hematoprotective effect: in the presence of OL9-116 and under combined treatment, leukocytosis was less pronounced and erythrocyte parameters showed more favorable dynamics than in groups receiving topotecan alone, with no evidence of hepato- or splenotoxicity based on liver and spleen weights.

It should be noted that the question of side effects associated with the potential interaction of substances remains unresolved. The structures of OL9-116 and topotecan do not contain functional groups capable of forming covalent bonds with each other (only hydroxyl groups, carbonyl groups, and carboxyl group derivatives are present). Should these molecules coexist in the same location at the same time in their unaltered state, non-covalent interactions could occur—interactions that cannot be detected by any analytical methods in in vivo experiments. Drug–drug interactions involve the interaction of substances with the enzymes that metabolize them, thereby accelerating or slowing down their metabolism. This is a complex problem that requires extensive research in the future.

Thus, OL9-116 is characterized by the absence of mutagenic and genotoxic effects in standard in vitro assays, does not exhibit epigenetic activity in the HeLa TI test system, displays a favorable pharmacokinetic profile following intraperitoneal administration, and exerts an enhancing effect on the antitumor and antimetastatic activity of topotecan in RShM-5 and LLC models. Taken together, these data support the consideration of OL9-116 as a promising adjuvant for combination chemotherapy based on TOP1 inhibitors, with a favorable tolerability profile.

Despite the substantial volume of preclinical data obtained from mouse models, translating these findings into human clinical trials remains a complex and challenging process. Notably, the concordance rate of positive results between animal and clinical studies is estimated at 86% [46]. Assessing the risk–benefit ratio is a crucial aspect. While preclinical data from mouse models often demonstrate pronounced effects, actual clinical outcomes—particularly regarding toxicity—can differ significantly due to interspecies biological differences, the limited heterogeneity of mouse populations, and methodological constraints. Although mouse models remain a vital stage of preclinical testing, their results must be interpreted with due regard for statistical data on translational inefficiency and ethical considerations. Future research could extend this work through the use of humanized and organoid models.

## Figures and Tables

**Table 1:** Results of mutagenicity testing of OL9-116 in *Salmonella typhimurium* tester strains TA98 and TA100.

Group	Compound	Dose, mol/Plate	Strain *S. typhimurium*							
			TA98				TA100			
			S9−		S9+		S9−		S9+	
			M ± m	Me/Mc	M ± m	Me/Mc	M ± m	Me/Mc	M ± m	Me/Mc
**Negative control**		**–**	19 ± 3	1	18 ± 2	1	91 ± 3	1	86 ± 7	1
**Positive control**	DDDTDP	6.7 × 10^−2^	1400 ± 100	73.7	–	–	–	–	–	–
	2-AF	1.1 × 10^−1^	–	–	1800 ± 400	101.3	–	–	900 ± 200	10.2
	BaP	4 × 10^−2^	–	–	510 ± 30	28.3	–	–	460 ± 20	5.4
	NaN3	6.8 × 10^−2^	–	–	–	–	2000 ± 300	21.5	–	–
**Experimental**	OL9-116	4.4 × 10^−9^	19 ± 5	1	20 ± 1	1.1	76 ± 6	0.8	73 ± 4	0.8
		4.4 × 10^−8^	17 ± 1	0.9	21 ± 3	1.2	71 ± 7	0.8	90 ± 20	1.1
		4.4 × 10^−7^	14 ± 3	0.7	20 ± 2	1.1	90 ± 10	0.9	69 ± 5	0.8

M ± m—mean number of revertants per plate ± standard deviation; Me/Mc—ratio of the number of revertants in the experimental group to that in the control. Color coding indicates the mutagenic activity of the compound in the assay: green—no mutagenic activity; red—mutagenic (positive control); white—not tested.

**Table 2:** Effects of the tested compounds on cell proliferation characteristics and genomic damage in human lymphocyte cultures.

Compound	Nuclei per Cell			CBPI	N2 with ≥1 MNed Cells per 2000 Cells	Frequency of MNed Cells (Cells with ≥1 MNi) per 1000 Cells	NPB	NBUD
	N1	N2	N ≥ 3					
**Donor 1 (male)**								
**DMSO**	349	150	75	1.523	23	11.5	1	2
**IFO**	284	140	83	1.604	81	40.5 **	25 **	45 **
**Dac**	250	155	87	1.669 **	74	37 **	45 **	48 **
**OL9-116 0.1 µM**	347	151	75	1.525	20	10	1	3
**OL9-116 1 µM**	352	148	74	1.516	22	11	-	2
**OL9-116 10 µM**	348	153	75	1.526	19	9.5	1	-
**OL9-116 100 µM**	342	152	75	1.531	24	12	2	-
**Donor 2 (female)**								
**DMSO**	350	149	69	1.51	21	10.5	2	2
**IFO**	293	135	90	1.609 *	92	46 **	32 **	51 **
**Dac**	261	144	88	1.649 **	83	41.5 **	49 **	67 **
**OL9-116 0.1 µM**	350	147	73	1.514	17	8.5	1	1
**OL9-116 1 µM**	349	153	75	1.53	21	10.5	1	2
**OL9-116 10 µM**	353	152	71	1.51	20	10	1	2
**OL9-116 100 µM**	351	150	70	1.508	19	9.5	-	1

* *p* < 0.05; ** *p* < 0.01, Pearson’s χ^2^ test.

## Data Availability

The original contributions presented in this study are included in the article/Supplementary Material. Further inquiries can be directed to the corresponding author.

## Supplementary Materials

The following supporting information can be downloaded at https://www.mdpi.com/article/10.3390/ijms27209266/s1.

## Abbreviations

The following abbreviations are used in this manuscript:

CBMN	cytokinesis-block micronucleus assay
CBPI	cytokinesis-block proliferation index
Dac	dacarbazine
GFP	green fluorescent protein
HeLa TI	HeLa trichostatin A-inducible cells
IFO	ifosfamide
LLC	mouse Lewis lung carcinoma
NBUD	nuclear buds
MN	micronuclei
NPB	nucleoplasmic bridges
RShM-5	mouse cervical cancer
TDP1	tyrosyl-DNA phosphodiesterase 1
TGI	tumor growth inhibition
TOP1	topoisomerase 1
TPC	topotecan
TSA	Trichostatin A

## References

[1] Marusyk A., Janiszewska M., Polyak K. (2020). Intratumor Heterogeneity: The Rosetta Stone of Therapy Resistance. Cancer Cell.

[2] Lovly C.M., Salama A.K.S., Salgia R. (2016). Tumor Heterogeneity and Therapeutic Resistance. Am. Soc. Clin. Oncol. Educ. Book Am. Soc. Clin. Oncol. Annu. Meet..

[3] Crucitta S., Cucchiara F., Mathijssen R., Mateo J., Jager A., Joosse A., Passaro A., Attili I., Petrini I., van Schaik R. (2022). Treatment-Driven Tumour Heterogeneity and Drug Resistance: Lessons from Solid Tumours. Cancer Treat. Rev..

[4] Ayoub N.M. (2021). Editorial: Novel Combination Therapies for the Treatment of Solid Cancers. Front. Oncol..

[5] Palmer A.C., Chidley C., Sorger P.K. (2019). A Curative Combination Cancer Therapy Achieves High Fractional Cell Killing through Low Cross-Resistance and Drug Additivity. eLife.

[6] Evseev V.N., Stolyarov V.I., Ledin E.V. (2025). Combination or Single-Agent Chemotherapy in Metastatic Breast Cancer. Malig. Tumours.

[7] Mokhtari R.B., Homayouni T.S., Baluch N., Morgatskaya E., Kumar S., Das B., Yeger H. (2017). Combination Therapy in Combating Cancer. Oncotarget.

[8] Duarte D., Vale N. (2022). Evaluation of Synergism in Drug Combinations and Reference Models for Future Orientations in Oncology. Curr. Res. Pharmacol. Drug Discov..

[9] Lehàr J., Krueger A.S., Avery W., Heilbut A.M., Johansen L.M., Price E.R., Rickles R.J., Short G.F., Staunton J.E., Jin X. (2009). Synergistic Drug Combinations Improve Therapeutic Selectivity. Nat. Biotechnol..

[10] Doroshow J.H., Simon R.M. (2017). On the Design of Combination Cancer Therapy. Cell.

[11] Kwak E.L., Clark J.W., Chabner B. (2007). Targeted Agents: The Rules of Combination. Clin. Cancer Res..

[12] Koldysheva E.V., Men’shchikova A.P., Lushnikova E.L., Popova N.A., Kaledin V.I., Nikolin V.P., Zakharenko A.L., Luzina O.A., Salakhutdinov N.F., Lavrik O.I. (2019). Antimetastatic Activity of Combined Topotecan and Tyrosyl-DNA Phosphodiesterase-1 Inhibitor on Modeled Lewis Lung Carcinoma. Bull. Exp. Biol. Med..

[13] Nikolin V.P., Popova N.A., Kaledin V.I., Luzina O.A., Zakharenko A.L., Salakhutdinov N.F., Lavrik O.I. (2021). The Influence of an Enamine Usnic Acid Derivative (a Tyrosyl-DNA Phosphodiesterase 1 Inhibitor) on the Therapeutic Effect of Topotecan against Transplanted Tumors in vivo. Clin. Exp. Metastasis.

[14] Comeaux E.Q., van Waardenburg R.C.A.M. (2014). Tyrosyl-DNA Phosphodiesterase I Resolves Both Naturally and Chemically Induced DNA Adducts and Its Potential as a Therapeutic Target. Drug Metab. Rev..

[15] Zakharenko A.L., Luzina O.A., Chepanova A.A., Dyrkheeva N.S., Salakhutdinov N.F., Lavrik O.I. (2023). Natural Products and Their Derivatives as Inhibitors of the DNA Repair Enzyme Tyrosyl-DNA Phosphodiesterase 1. Int. J. Mol. Sci..

[16] Zakharenko A., Luzina O., Koval O., Nilov D., Gushchina I., Dyrkheeva N., Švedas V., Salakhutdinov N., Lavrik O. (2016). Tyrosyl-DNA Phosphodiesterase 1 Inhibitors: Usnic Acid Enamines Enhance the Cytotoxic Effect of Camptothecin. J. Nat. Prod..

[17] Okhina A.A., Kornienko T.E., Rogachev A.D., Luzina O.A., Popova N.A., Nikolin V.P., Zakharenko A.L., Dyrkheeva N.S., Pokrovsky A.G., Salakhutdinov N.F. (2023). Pharmacokinetic Study of Tdp1 Inhibitor Resulted in a Significant Increase in Antitumor Effect in the Treatment of Lewis Lung Carcinoma in Mice by Its Combination with Topotecan. J. Pharm. Biomed. Anal..

[18] Okhina A.A., Kornienko T.E., Rogachev A.D., Luzina O.A., Popova N.A., Nikolin V.P., Zakharenko A.L., Dyrkheeva N.S., Pokrovsky A.G., Salakhutdinov N.F. (2025). Tissue Distribution of OL9-116, a Tdp1 Inhibitor Based on Usnic Acid, Is Significantly Altered in Lewis Lung Carcinoma-Bearing Mice Compared to Healthy Animals. J. Pharm. Biomed. Anal..

[19] OECD (2023). Test No. 487: In Vitro Mammalian Cell Micronucleus Test.

[20] Thomas P., Fenech M., Didenko V.V. (2011). Cytokinesis-Block Micronucleus Cytome Assay in Lymphocytes. DNA Damage Detection In Situ, Ex Vivo, and In Vivo: Methods and Protocols.

[21] Sasaki K., Saito K., Kaneko S., Kawai Y. (2019). Detection Method for Chromosomal Abnormalities and Method for Evaluating Inducibility. U.S. Patent.

[22] Maksimova V., Shalginskikh N., Vlasova O., Usalka O., Beizer A., Bugaeva P., Fedorov D., Lizogub O., Lesovaya E., Katz R. (2021). HeLa TI Cell-Based Assay as a New Approach to Screen for Chemicals Able to Reactivate the Expression of Epigenetically Silenced Genes. PLoS ONE.

[23] Maksimova V., Popova V., Kholodova A., Makus J., Usalka O., Lylova E., Kudriashov A., Belitsky G., Yakubovskaya M., Kirsanov K. (2026). Epigenetic Activity of Cancer Therapy Drugs Revealed by HeLa TI Cell-Based Assay. Epigenomes.

[24] Katz R.A., Jack-Scott E., Narezkina A., Palagin I., Boimel P., Kulkosky J., Nicolas E., Greger J.G., Skalka A.M. (2007). High-Frequency Epigenetic Repression and Silencing of Retroviruses Can Be Antagonized by Histone Deacetylase Inhibitors and Transcriptional Activators, but Uniform Reactivation in Cell Clones Is Restricted by Additional Mechanisms. J. Virol..

[25] ICH S9 Non-Clinical Evaluation for Anticancer Pharmaceuticals—Scientific Guideline|European Medicines Agency (EMA). https://www.ema.europa.eu/en/ich-s9-non-clinical-evaluation-anticancer-pharmaceuticals-scientific-guideline.

[26] Tryakin A.A., Besova N.S., Volkov N.M., Gladkov O.A., Karaseva V.V., Sakaeva D.D., Stradaeva I.Y., Fadeeva N.V. (2021). Practical recommendations on the general principles of antitumor drug therapy. Malig. Tumors.

[27] Shekunova E.V., Kovaleva M.A., Makarova M.N., Makarov V.G. (2020). Dose Selection in Preclinical Studies: Cross-Species Dose Conversion. Bull. Sci. Cent. Expert Eval. Med. Prod..

[28] Langenau D.M., Sweet-Cordero A., Wechsler-Reya R., Dyer M.A. (2015). Preclinical Models Provide Scientific Justification and Translational Relevance for Moving Novel Therapeutics into Clinical Trials for Pediatric Cancer. Cancer Res..

[29] Guichard S., Montazeri A., Chatelut E., Hennebelle I., Bugat R., Canal P. (2001). Schedule-Dependent Activity of Topotecan in OVCAR-3 Ovarian Carcinoma Xenograft: Pharmacokinetic and Pharmacodynamic Evaluation. Clin. Cancer Res..

[30] Chen J., Lu Q., Balthasar J.P. (2007). Mathematical Modeling of Topotecan Pharmacokinetics and Toxicodynamics in Mice. J. Pharmacokinet. Pharmacodyn..

[31] Simon Davis D.A., Ritchie M., Hammill D., Garrett J., Slater R.O., Otoo N., Orlov A., Gosling K., Price J., Yip D. (2023). Identifying Cancer-Associated Leukocyte Profiles Using High-Resolution Flow Cytometry Screening and Machine Learning. Front. Immunol..

[32] Demers M., Suidan G.L., Andrews N., Martinod K., Cabral J.E., Wagner D.D. (2018). Solid Peripheral Tumor Leads to Systemic Inflammation, Astrocyte Activation and Signs of Behavioral Despair in Mice. PLoS ONE.

[33] Tazetdinova A.A., Luzina O.A., Polovinka M.P., Salakhutdinov N.F., Tolstikov G.A. (2009). Amino-Derivatives of Usninic Acid. Chem. Nat. Compd..

[34] (2006). Biological Evaluation of Medical Devices—Part 3: Evaluation of Genotoxicity, Carcinogenicity, Reproductive Toxicity and Developmental Toxicity.

[35] Maron D.M., Ames B.N. (1983). Revised Methods for the Salmonella Mutagenicity Test. Mutat. Res. Mutagen. Relat. Subj..

[36] Belitskiĭ G.A., Fonshteĭn L.M., Khudoleĭ V.V., Demakov V.A., Kiseleva M.G. (1987). Sovol as an inducer of procarcinogen activating microsomal enzymes. Eksp. Onkol..

[37] Sioen S., Cloet K., Vral A., Baeyens A. (2020). The Cytokinesis-Block Micronucleus Assay on Human Isolated Fresh and Cryopreserved Peripheral Blood Mononuclear Cells. J. Pers. Med..

[38] Bolognesi C., Fenech M. (2013). Micronucleus Assay in Human Cells: Lymphocytes and Buccal Cells. Genotoxicity Assessment.

[39] Fenech M., Chang W.P., Kirsch-Volders M., Holland N., Bonassi S., Zeiger E. (2003). HUMN Project: Detailed Description of the Scoring Criteria for the Cytokinesis-Block Micronucleus Assay Using Isolated Human Lymphocyte Cultures. Mutat. Res. Toxicol. Environ. Mutagen..

[40] Costa S., Ceppi M., Costa C., Silva S., Pereira C., Laffon B., Bonassi S., Teixeira J.P. (2016). The Cytokinesis-Block Micronucleus (CBMN) Assay in Human Populations Exposed to Styrene: A Systematic Review and Meta-Analysis. Mutat. Res. Mutat. Res..

[41] Bonassi S., Znaor A., Ceppi M., Lando C., Chang W.P., Holland N., Kirsch-Volders M., Zeiger E., Ban S., Barale R. (2007). An Increased Micronucleus Frequency in Peripheral Blood Lymphocytes Predicts the Risk of Cancer in Humans. Carcinogenesis.

[42] Mironov A.N. (2012). Guidelines for Conducting Preclinical Studies of Drugs; Part One.

[43] Solodovnikov A.G., Morkovin E.I., Kurkin D.V., Sorokina E.Y., Peretolchina T.F. (2022). Considerations for permitted daily exposure calculation for contaminants in medicinal products manufactured in shared facilities. Bull. Sci. Cent. Expert Eval. Med. Prod. Regul. Res. Med. Eval..

[44] Zhang Y., Huo M., Zhou J., Xie S. (2010). PKSolver: An Add-in Program for Pharmacokinetic and Pharmacodynamic Data Analysis in Microsoft Excel. Comput. Methods Programs Biomed..

[45] Abo-EL-Sooud K., Hock F., Gralinski M. (2020). Absolute and Relative Bioavailability. Drug Discovery and Evaluation: Methods in Clinical Pharmacology.

[46] Ineichen B.V., Furrer E., Grüninger S.L., Zürrer W.E., Macleod M.R. (2024). Analysis of animal-to-human translation shows that only 5% of animal-tested therapeutic interventions obtain regulatory approval for human applications. PLoS Biol..

