# Supplementary material for: Safety and Antitumor Activity of the Usnic Acid Derivative OL9-116, a TDP1 Inhibitor, as an Adjunctive Therapy in Combination with Topotecan
Source: Int J Mol Sci. 2026 Oct 19;27(20):9266. doi: 10.3390/ijms27209266 (PMC13704960; doi:10.3390/ijms27209266)
Supplement: Supplementary file 1 [file ijms-27-09266-s001.zip › ijms-4559882-supplementary.pdf]

Supplementary Materials

Safety and Antitumor Activity of the Usnic Acid Derivative OL9-116, a TDP1 Inhibitor, as an Adjunctive Therapy in Combination with Topotecan

Tatyana E. Kornienko [1], Alexandra L. Zakharenko [1,*], Irina A. Chernyshova [1,2], Arina A. Chepanova [1,2], Nadezhda S. Dyrkheeva [1], Alina A. Firsova [3,4], Artem D. Rogachev [3,4], Olga A. Luzina [3], Nelly A. Popova [2,5], Varvara P. Maksimova [6], Olga A. Vlasova [6], Valentina P. Deryagina [6], Alexander Ya. Rodnyy [2], Nariman F. Salakhutdinov [3] and Olga I. Lavrik [1,5,7]

[1] Knorre Institute of Chemical Biology and Fundamental Medicine, Siberian Branch of the Russian Academy of Sciences, 630090 Novosibirsk, Russia; t.kornienko1995@gmail.com (T.E.K.); arinachepanova@mail.ru (A.A.C.)
[2] Federal Research Center Institute of Cytology and Genetics, Siberian Branch of the Russian Academy of Sciences, 10, Akademika Lavrentieva Ave., 630090 Novosibirsk, Russia
[3] N. N. Vorozhtsov Novosibirsk Institute of Organic Chemistry, Siberian Branch of the Russian Academy of Sciences, 9, Akademika Lavrentieva Ave., 630090 Novosibirsk, Russia; rogachev@nioch.nsc.ru (A.D.R.); luzina@nioch.nsc.ru (O.A.L.)
[4] Institute of Medicine and Medical Technologies, Novosibirsk State University, Pirogov St., 2, 630090 Novosibirsk, Russia
[5] Department of Natural Sciences, Novosibirsk State University, Pirogov St., 2, 630090 Novosibirsk, Russia
[6] N.N. Blokhin National Medical Research Center of Oncology, 24 Kashirskoe Shosse, 115478 Moscow, Russia
[7] The Institute of Biology and Biotechnology, Altai State University, Pr. Lenina 61, 656049 Barnaul, Russia
* Correspondence: zakharenko@1bio.ru

Table S1

Pharmacokinetic parameters (mean ± SD) of the agent OL9-116 after intraperitoneal administration to mice at the dose of 5 mg/kg.

Parameter	Calculated value
$T_{1/2}$, h	8.6 ± 4.8
T_{max}, h	2.2 ± 0.8
C_{max}, ng/mL	4417 ± 3040
$AUC_{0\text{-}\infty}$, ng/mL×h	$(6.\ 4 \pm 2.3)\times10^{4}$
$MRT_{0\text{-}\infty}$, h	12.9 ± 7.2
CL, (mg/kg)/(ng/mL)/h	$(2.0 \pm 1.1)\times10^{-4}$

$T_{1/2}$—half-life, T_{max}—time to maximum concentration, C_{max}—maximum concentration, $AUC_{0\text{-}\infty}$—area under the plasma drug concentration–time curve from zero point up to infinity, $MRT_{0\text{-}\infty}$—mean time a molecule resides in the body, CL—total clearance of drug from the blood.

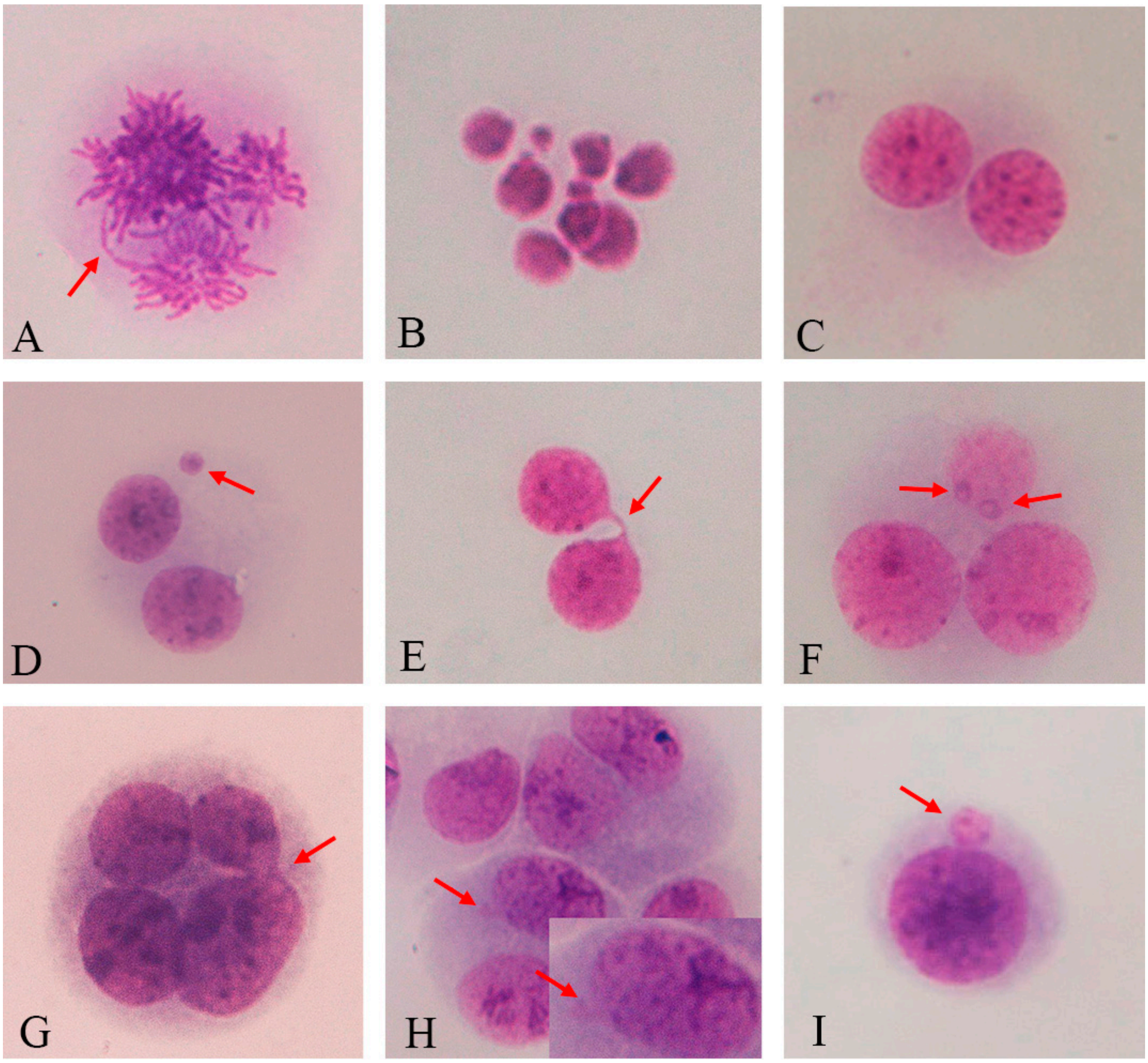

Figure S1. DNA damage and cytotoxicity biomarkers in the cytokinesis-block micronucleus (CBMN) assay in human peripheral blood lymphocytes: (A) trinucleated cell in mitosis with cytoplasmic bridges; (B) apoptotic cell; (C) binucleated cell; (D) binucleated cell with a micronucleus; (E) binucleated cell with a cytoplasmic bridge; (F) trinucleated cell with micronuclei; (G) tetranucleated cell with a cytoplasmic bridge; (H) binucleated cell with a nuclear bud; (I) mononucleated cell with a micronucleus.

Table S2. Time course of changes in leukocyte concentration (thousands/μL) in CBA/Lac mice bearing the transplanted RShM-5 tumor model.

	0.9% NaCl	15% DMSO + 10% Tween-80	TPC 1 mg/kg x1	TPC 0.2 mg/kg x5	TPC 0.07 mg/kg x5	OL9-116 5 mg/kg i/p x1	OL9-116 1 mg/kg i/p x5	OL9-116 150 mg/kg i/g x1	TPC 1 mg/kg + OL9-116 5 mg/kg i/p x1	TPC 0.2 mg/kg + OL9-116 1 mg/kg i/p x5	TPC 0.07 mg/kg + OL9-116 1 mg/kg i/p x5	TPC 1 mg/kg + OL9-116 150 mg/kg i/g x1
Before tumor transplantation	10.6±0.8	10.7±1	10.3±1	9.2±1.2	10.2±1.1	9.8±1	9.3±1.2	10.9±1	9.1±1.5	9.5±1.5	9±1.6	8.9±1.2
With a tumor (before treatment)	12.2±1.2	11.8±1.3	11±1.3	9.6±1.3	10.9±1.2	10.4±1.1	9.9±1	12.2±1.4	9.6±1.5	10.3±2.1	9.8±2.1	9.3±1.3
With a tumor (after treatment)	12.8±1.1	12.4±1	11.4±1.2	10±1.3	11.3±1.2	10.7±1.3	10.2±1.2	12.3±1.4	9.5±1.4	9.7±1.8	9.2±1.7	9.4±1.3
End of the experiment	12.9±1	13.9±2	11.6±1	10.4±1.2	11.9±1.2	10.9±1.3	10.5±1.3	12.5±1.3	9.4±1.4	9.5±1.6	8.91±1.6	9.3±1.1

Table S3. Time course of changes in erythrocyte concentration (million/µL) in CBA/Lac mice bearing the transplanted RShM-5 tumor model.

	0.9% NaCl	15% DMSO + 10% Tween-80	TPC 1 mg/kg x1	TPC 0.2 mg/kg x5	TPC 0.07 mg/kg x5	OL9-116 5 mg/kg i/p x1	OL9-116 1 mg/kg i/p x5	OL9-116 150 mg/kg i/g x1	TPC 1 mg/kg + OL9-116 5 mg/kg i/p x1	TPC 0.2 mg/kg+ OL9-116 1 mg/kg i/p x5	TPC 0.07 mg/kg + OL9-116 1 mg/kg i/p x5	TPC 1 mg/kg + OL9-116 150 mg/kg i/g x1
Before tumor transplantation	8.4±0.3	8.1±0.1	8.2±0.2	8.4±0.2	8.4±0.2	8.4±0.1	8.5±0.2	8.5±0.2	8.4±0.3	8.4±0.2	8.1±0.2	8±0.8
With a tumor (before treatment)	8±0.2	8±0.2	7.9±0.2	8.2±0.2	8.2±0.1	8.1±0.1	8.3±0.1	8.3±0.3	8.3±0.3	8.3±0.3	7.9±0.2	7.5±1.1
With a tumor (after treatment)	7.7±0.4	7.7±0.3	7.8±0.2	8±0.2	8.1±0.1	8.1±0.1	8.2±0.1	8.2±0.2	8.4±0.3	8.3±0.2	8.1±0.2	7.6±1.1
End of the experiment	7.4±0.4	7.6±0.3	7.8±0.2	7.9±0.2	8±0.1	8±0.1	8.2±0.1	8.1±0.2	8.4±0.4	8.4±0.2	8.1±0.2	7.7±1

Table S4. Time course of changes in leukocyte concentration (thousand/µL) in C57BL/6J mice bearing the transplanted LLC tumor model.

	0.9% NaCl	15% DMSO + 10% Tween-80	OL9-116 1 mg/kg i/p x5	TPC 0.2 mg/kg x5	TPC 0.07 mg/kg x5	TPC 0.2 mg/kg + OL9-116 1 mg/kg i/p x5	TPC 0.07 mg/kg + OL9-116 1 mg/kg i/p x5
Before tumor transplantation	9.4±0.9	8.9±0.7	8.7±1	8.5±1.1	9.4±1.1	7.9±0.8	8.4±0.8
With a tumor (before treatment)	11.8±1.4	10.8±0.7	10.1±1	11.4±1.3	12.2±1.8	9.4±0.9	8.8±1
With a tumor (after treatment)	13.5±1.7	11.5±1	9.8±1.2	12.4±1.6	12.8±2.1	9±1.1	8.7±0.9
End of the experiment	14.1±1.9	12.2±0.8	9.8±1.2	13.3±1.7	13.7±3.3	8.7±1.2	8.9±1.1

Table S5. Time course of changes in erythrocyte concentration (million/µL) in C57BL/6J mice bearing the transplanted LLC tumor model.

	0.9% NaCl	15% DMSO + 10% Tween-80	OL9-116 1 mg/kg i/p x5	TPC 0.2 mg/kg x5	TPC 0.07 mg/kg x5	TPC 0.2 mg/kg + OL9-116 1 mg/kg i/p x5	TPC 0.07 mg/kg + OL9-116 1 mg/kg i/p x5
Before tumor transplantation	7.1±0.5	6.9±0.5	7.2±0.4	6.9±0.5	7±0.6	7±0.4	7±0.7
With a tumor (before treatment)	6.5±0.6	6.6±0.3	6.6±0.5	6.3±0.4	6.4±0.6	6.3±0.4	6.4±0.8
With a tumor (after treatment)	5.9±0.5	5.9±0.2	6.8±0.4	5.8±0.4	6±0.4	6.2±0.5	6.5±0.8
End of the experiment	5.3±0.4	5.1±0.5	7±0.4	5.5±0.2	5.6±0.5	6.3±0.6	6.6±0.8
